# An Approach to Study Species Persistence in Unconstrained Random Networks

**DOI:** 10.1038/s41598-019-50373-z

**Published:** 2019-10-01

**Authors:** Samuel M. Fischer, Andreas Huth

**Affiliations:** 1 Department of Mathematical and Statistical Sciences, University of Alberta, Edmonton, AB T6G 2G1 Canada; 2 UFZ - Helmholtz Centre for Environmental Research, Department of Ecological Modelling, Permoserstraße 15, 04318 Leipzig, Germany; 3 Institute of Environmental Systems Research, Osnabrück University, Barbarastraße 12, 49076 Osnabrück, Germany; 4 iDiv - German Centre for Integrative Biodiversity Research Halle-Jena-Leipzig, Deutscher Platz 5e, 04103 Leipzig, Germany

**Keywords:** Ecological modelling, Ecological networks, Theoretical ecology

## Abstract

The connection between structure and stability of ecological networks has been widely studied in the last fifty years. A challenge that scientists continue to face is that in-depth mathematical model analysis is often difficult, unless the considered systems are specifically constrained. This makes it challenging to generalize results. Therefore, methods are needed that relax the required restrictions. Here, we introduce a novel heuristic approach that provides persistence estimates for random systems without limiting the admissible parameter range and system behaviour. We apply our approach to study persistence of species in random generalized Lotka-Volterra systems and present simulation results, which confirm the accuracy of our predictions. Our results suggest that persistence is mainly driven by the linkage density, whereby additional links can both favour and hinder persistence. In particular, we observed “persistence bistability”, a rarely studied feature of random networks, leading to a dependency of persistence on initial species densities. Networks with this property exhibit tipping points, in which species loss can lead to a cascade of extinctions. The methods developed in this paper may facilitate the study of more general models and thereby provide a step forward towards a unifying framework of network architecture and stability.

## Introduction

The study of ecosystem stability has attracted researchers’ interest for many years^1^. Since May’s influential paper^2^, a large body of studies have revealed a variety of mechanisms affecting ecosystem stability^3^. Such analyses are key to our understanding of how changes, for example induced by climate change, land use, or invasive species, affect species richness and services of ecosystems.

As experiments on ecological networks are costly and limited in scale, many theoretical studies consider randomly generated ecological networks^1,3^. Thereby, researchers use a toolset reaching from analytical techniques applied to abstract models^2,4,5^ to simulation approaches considering ecological networks with architectures matching empirical observations^6–8^. While more complex models may be better suited to accurately predict the behaviour of real ecosystems, abstract and simplified models often allow an in-depth mathematical analysis, which in turn can yield a more comprehensive understanding of the principles governing ecosystem stability. The results obtained with analytical approaches, however, can be limited by restrictions imposed on the model to make the mathematical analysis feasible. Therefore, efforts have been made to apply analytical techniques to more intricate models and to relax simplifying assumptions^9–11^.

Nonetheless, akin to the importance of considering ecologically informed models, it is relevant to assess stability with a criterion strongly linked to ecological questions. Many theoretical studies consider asymptotic stability, the ability of a system to return to a state after a (small) perturbation^2,4,7^. This stability measure describes well how a system reacts to changes, but other approaches can also provide information about the system’s *state* before or after a disturbance. For that reason, “persistence”^12^, quantifying how many species coexist in an ecosystem for the long term, is actively studied^5,8,13–18^.

A challenge associated with the study of persistence is that the transient system behaviour can affect how many species persist. Depending on the initial condition or the nature of a perturbation, a system may approach different states with different sets and numbers of persisting species. Since it is difficult to consider transient behaviour with analytical methods, studies on persistence often use either simulation approaches^8,13,14,19,20^ or consider constrained networks in which a unique set of persisting species can be identified^5,16,21^. Both methods, however, may limit the generality of the results. Consequently, an approach would be desirable that both maintains the advantages of analytical techniques while also allowing the study of a broad spectrum of systems. This will be the subject of this paper.

To circumvent the restrictions associated both with simulation methods and the classical analytical techniques, we propose a middle way: based on approximations, we derive a heuristic for persistence of species in random networks. We test the validity of our estimates with simulations. This allows us both to study the mechanisms behind persistence and to predict possible system behaviour without the need of simulating each system of interest. To demonstrate the potential of our approach, we develop our heuristic for randomly generated generalized Lotka-Volterra systems and examine the role of (1) species richness, (2) connectivity, (3) distribution of intrinsic growth/death rates, (4) distribution of interaction parameters, and (5) the initial densities of species. Thereby, we minimize the effect of model restrictions on our results by ensuring that the considered models are able to show a wide range of qualitatively different system behaviour.

Our results suggest that persistence is mainly driven by the linkage density, i.e. the average number of links per species. Thereby, addition of links can have both a stabilizing or a destabilizing effect, depending on the distribution of the intrinsic growth or death rates and the interaction parameters. Intermediate linkage densities can lead to local persistence maxima and minima. Furthermore, we identify bistable systems, in which the number of coexisting species depends on the initial species densities. These bistable systems have threshold values so that addition or removal of links could either lead to persistence of most species or a collapse of the system.

This paper is structured as follows: first we explain the base model of our study. Then we derive a heuristic estimate for the proportion of species that coexist in a system. In a next step, we briefly describe the simulation approach that we used to assess the accuracy of our estimates and present the simulation results. Lastly, we discuss the limitations of our approach and its contributions to the current debate on ecosystem stability.

## Methods

In this study, we consider generalized Lotka-Volterra systems:

1 $$\frac{d{x}_{i}}{dt}={x}_{i}({r}_{i}+\sum _{j\in I}\,{a}_{ji}{x}_{j})$$

with $i\in I=\{1,\ldots ,n\}$. If the intrinsic growth rate is positive, i.e. ${r}_{i} > 0$, then species *i* can grow independently of other species. We therefore call it a “producer”. If ${r}_{i} < 0$, then species *i* cannot survive without interactions with other species. Hence, we call it a “consumer”. The parameters *a*_*ji*_ describe the interactions between species $j\in I$ and species *i*. The model allows mutualistic, competitive, and predator-prey interactions.

Typically, not all species interact with each other in real ecosystems. Therefore, it is reasonable to set some interaction parameters *a*_*ji*_ to 0. The fraction of existing interactions in relation to the total number of species pairs is called the connectivity *c* of the network.

The Lotka-Volterra system (1) may permit unbounded growth of species. To keep species densities from diverging, we introduce ‘capacities’ *K*_*i*_ at which species stop growing:

2 $$\frac{d{x}_{i}}{dt}=\{\begin{array}{cc}{x}_{i}({r}_{i}+\sum _{j\in I}\,{a}_{ji}{x}_{j}) & {\rm{i}}{\rm{f}}\,{x}_{i} < {K}_{i}\,{\rm{o}}{\rm{r}}\,{r}_{i}+\sum _{j\in I}\,{a}_{ji}{x}_{j} < 0\\ 0 & {\rm{e}}{\rm{l}}{\rm{s}}{\rm{e}}\end{array}.$$

The bounds *K*_*i*_ can be seen as modelling a strongly non-linear intra-specific competition mechanism. In contrast to choosing large intra-specific competition constants *a*_*ii*_, the sharp bound does not preclude complex system behaviour found in natural systems, such as multistabilty, oscillations, and chaotic dynamics.

It would as well be possible to bound species densities by introducing functional response interaction terms. This, however, would require us to specify a trophic network structure before the individual interaction parameters are determined. While possible in principle, this requirement makes it considerably more difficult to describe the network generation process in simple mathematical terms. We therefore work with the simple model here.

Rescaling the species densities allows us to set the capacity ${K}_{i}=1$ for all species. This also affects the distribution of the model parameters. However, we will argue later that the choice of the capacities has minor influence on persistence of species. Therefore, we will progress with ${K}_{i}=1$ for increased simplicity.

System (2) has a discontinuous right hand side. This can affect existence and uniqueness of solutions^22,23^. Nonetheless, in Supplementary Appendix A, we show that system (2) with ${K}_{i}=1$ has a unique solution for all initial conditions ${\bf{x}}(0)={{\bf{x}}}_{0}\in {[0,1]}^{n}$.

To examine the relationship between network structure and persistence of species, we consider systems with random parameter values. We assume the following:

1. The intrinsic growth constants *r*_*i*_ are drawn from a normal distribution with mean *μ*_*r*_ and variance ${\sigma }_{r}^{2}$.
2. The entries *a*_*ji*_ of the interaction matrix are drawn from a normal distribution with mean *μ*_*a*_ and variance ${\sigma }_{a}^{2}$.
3. Each link exists with probability *c*. That is, ${\mathbb{P}}({a}_{ji}\ne 0)=c$.
4. The values of the intra-specific interaction constants ${a}_{ii} < 0$ are either drawn from a normal distribution with variance ${\sigma }_{a}^{2}$ centered and truncated at 0 or chosen deterministically as ${a}_{ii}=d$.
5. The initial population densities are drawn such that a constant *b* determines the proportion of “native” species with high initial densities to “invaders” with low initial densities. For the first $b\cdot n$ species ($b\cdot n$ rounded to the closest integer), ${x}_{i}(0)$ follows a uniform distribution on $(0,1]$, and for the remaining species, ${x}_{i}(0)$ follows a uniform distribution on $(0,\varepsilon ]$. Thereby, the constant $\varepsilon \ll 1$ is small.

All parameters are independently drawn from their respective distributions. The species richness *n*, the connectivity *c*, and the parameters *μ*_*r*_, ${\sigma }_{r}^{2}$, *μ*_*a*_, and ${\sigma }_{a}^{2}$ give rise to a family of comunity models. Our goal is to examine how the choice of these parameters affects the expected fraction *P* of coexistent species and how this quantity depends on the initial condition.

There are different definitions of persistence^24^. In this paper, we say a species *i* persists if it has a positive long-time average density: $\mathop{\mathrm{lim}\,{\rm{\inf }}}\limits_{T\to \infty }\frac{1}{T}\,{\int }_{0}^{T}\,{x}_{i}(t)dt > 0$. This persistence definition is slightly stronger than the weak persistence in ref.^24^.

In Table 1, we provide a list of all the parameters and symbols used in this paper.

**Table 1:** Definitions and explanations of symbols and parameters used in this paper.

Symbol	Definition in mathematical terms	Definition in words
*n*		Species richness / dimension of the dynamical system
*c*	${\mathbb{P}}({a}_{ji}\ne 0)$ for any *i*, $j\in I$, $i\ne j$	Connectivity of the network / expected fraction of existing links
*l*	$n(c-1)$	Linkage density / number of links per species
*I*	$\{1,\ldots ,n\}$	Set of species indices
*I*_*i*_	$I\backslash \{i\}$	Set of species indices excluding species *i*
*r*_*i*_		Intrinsic growth/death rate of species *i*
*a*_*ji*_		Interaction parameter modelling the impact of species *j* on species *i*
*K*_*i*_		Capacity of species *i*
${x}_{i}(t)$		Density of species *i* at time *t*
${g}_{i}(t)$	${r}_{i}+\sum _{j\in {I}_{i}}\,{a}_{ji}{x}_{j}(t)$	Non-truncated per-capita growth rate of species *i* at time *t*
${\bar{x}}_{i}$	$\mathop{\mathrm{lim}}\limits_{T\to \infty }\frac{1}{T}\,{\int }_{0}^{T}\,{x}_{i}(t)dt$	Average density of species *i*
${\bar{g}}_{i}$	$\mathop{\mathrm{lim}}\limits_{T\to \infty }\frac{1}{T}\,{\int }_{0}^{T}\,{g}_{i}(t)dt={r}_{i}+\sum _{j\in {I}_{i}}\,{a}_{ji}{\bar{x}}_{j}$	Average non-truncated per-capita growth rate of species *i*
*μ*_*r*_	${\mathbb{E}}({r}_{i})$ for any $i\in I$	Mean intrinsic growth/death rate
${\sigma }_{r}^{2}$	${\mathbb{V}}({r}_{i})$ for any $i\in I$	Variance of the intrinsic growth/death rate
*μ*_*a*_	${\mathbb{E}}({a}_{ji}|{a}_{ji}\ne 0)$ for any *i*, $j\in I$, $i\ne j$	Mean strength of existing links
${\sigma }_{a}^{2}$	${\mathbb{V}}({a}_{ji}|{a}_{ji}\ne 0)$ for any *i*, $j\in I$, $i\ne j$	Variance of the strengths of existing links
*μ*_*g*_	${\mathbb{E}}({\bar{g}}_{i})$ for any $i\in I$	Mean of the average non-truncated per-capita growth rate
${\sigma }_{g}^{2}$	${\mathbb{V}}({\bar{g}}_{i})$ for any $i\in I$	Variance of the average non-truncated per-capita growth rate
*μ*_*x*_	${\mathbb{E}}({\bar{x}}_{i}|{\bar{x}}_{i} > 0)$ for any $i\in I$	Mean of the average density of surviving species
${\sigma }_{x}^{2}$	${\mathbb{V}}({\bar{x}}_{i}|{\bar{x}}_{i} > 0)$ for any $i\in I$	Variance of the average density of surviving species
*n*_*P*_	${\mathbb{E}}(|\{i\in I:{\bar{x}}_{i} > 0\}|)$	Expected number of persisting species
*P*	$\frac{{n}_{P}}{n}={\mathbb{P}}({\bar{x}}_{i} > 0)$ for any $i\in I$	Expected fraction of persisting species
N(*μ*, *σ*^2^)		Normal distribution with mean *μ* and variance *σ*^2^
${S}_{{\rm{N}}}(x;\mu ,{\sigma }^{2})$	$\frac{1}{\sqrt{2\pi }\sigma }\,{\int }_{x}^{\infty }\,{e}^{\frac{{(t-\mu )}^{2}}{2{\sigma }^{2}}}dt$	Survival function of a normal distribution with mean *μ* and variance *σ*^2^
*b*		Fraction of species with high initial density
$\varepsilon$		Extinction threshold / maximal initial density of species with low initial density
*T*		Time horizon of simulations

### Basic assumption and observations

Our goal is to estimate the expected fraction *P* of species that coexist in a system. To write *P* as function of the model parameters, we apply a novel heuristic approach. We argue that the heuristic provides a valuable approximation that deepens our understanding of persistence in ecological networks.

Let us start by considering the average population density

3 $${\bar{x}}_{i}:=\mathop{\mathrm{lim}}\limits_{T\to \infty }\frac{1}{T}\,{\int }_{0}^{T}\,{x}_{i}(t)dt.$$

This limit exists in all systems that approach steady states or limit cycles, and even in some systems with chaotic dynamics. Nonetheless, there may be systems (2), in which the limit does not exist. We neglect these cases by assuming the following:

#### **Assumption**.

*The all-time average population density* ${\bar{x}}_{i}$ *exists for all species i*.

It is intuitive that persistence of a species is linked to its per-capita growth rate. To make this link explicit, let us define the non-trunctated per-capita growth rate *g*_*i*_ of species *i* as

4 $${g}_{i}({\bf{x}}):={r}_{i}+\sum _{j\in {I}_{i}}\,{a}_{ji}{x}_{j}$$

with ${I}_{i}:=I\backslash \{i\}$. Here, we ignore the discontinuity of the original equation (2) and the self-damping term ${a}_{ii}{x}_{i}$, which does not affect the survival of species *i*.

Now we consider the long-time average of *g*_*i*_. If ${\bar{x}}_{i}$ exists, the average non-trunctated per-capita growth rate

5 $${\bar{g}}_{i}:={r}_{i}+\sum _{j\in {I}_{i}}\,{a}_{ji}{\bar{x}}_{j}$$

exists as well. This quantity provides us with a useful characterization of persistent species. In Supplementary Appendix B, we show that a species persists if and only if ${\bar{g}}_{i} > 0$. The intuition behind the proof is that intra-specific competition cannot drive a population to extinction if the population would grow on average otherwise. In the opposite case, the population declines to zero in the limit.

Now suppose we picked a species *i* from the species pool *I*. Since the probability that species *i* persists is equal to the expected fraction *P* of coexisting species, we can write *P* as a probability:

6 $$P={\mathbb{P}}({\bar{g}}_{i} > 0)={\mathbb{P}}({r}_{i}+\sum _{j\in {I}_{i}}\,{a}_{ji}{\bar{x}}_{j} > 0).$$

This equation is the basis of our heuristic.

### Heuristic derivation

We proceed by deriving a formula for the right hand side of Eq. (6), which gives us an expression for the expected fraction *P* of persisting species. To do this, we need to approximate the distribution of ${\bar{g}}_{i}$. If species *i* is not affected by any other surviving species, i.e. ${\sum }_{j\in {I}_{i}}{a}_{ji}{\bar{x}}_{j}=0$, then ${\bar{g}}_{i}$ follows the same distribution as the growth constants *r*_*i*_. This case is most prevalent in small or sparse networks or in networks in which only few species survive. In large and dense networks, however, it is likely that each species *i* is affected by some other species, and ${\bar{g}}_{i}$ depends on the distribution of the interaction constants. We will focus on this case first and refine our result in the next section by incorporating the possibility that some species are not affected by other species.

We start approximating the distribution of ${\bar{g}}_{i}$ by estimating its mean *μ*_*g*_ and variance ${\sigma }_{g}^{2}$. Let *μ*_*x*_ and ${\sigma }_{x}^{2}$ be the (unknown) conditional mean and variance of ${\bar{x}}_{j}$ given that species *j* persists. Then *μ*_*g*_ is given by

7 $$\begin{array}{rcl}{\mu }_{g} & = & {\mathbb{E}}({r}_{i}+\sum _{j\in {I}_{i}}\,{a}_{ji}{\bar{x}}_{j})={\mathbb{E}}({r}_{i})+(n-1)\,{\mathbb{E}}({a}_{ji}{\bar{x}}_{j})\\  & = & {\mu }_{r}+(n-1)\,c{\mu }_{a}P{\mu }_{x}+(n-1)\,{\rm{Cov}}({a}_{ji},{\bar{x}}_{j}).\end{array}$$

Here, we used that

8 $${\mathbb{E}}({a}_{ji})={\mathbb{P}}({a}_{ji}\ne 0)\,{\mathbb{E}}({a}_{ji}|{a}_{ji}\ne 0)=c{\mu }_{a}$$

9 $${\mathbb{E}}({\bar{x}}_{j})={\mathbb{P}}({\bar{x}}_{j} > 0)\,{\mathbb{E}}({\bar{x}}_{j}|{\bar{x}}_{j} > 0)=P{\mu }_{x}.$$

In Supplementary Appendix C, we show that the covariance between *a*_*ji*_ and ${\bar{x}}_{j}$ is bounded by a term proportional to $\frac{1}{\sqrt{n}}$. Our proof exploits that the covariance matrix for the *a*_*ji*_ and ${\bar{x}}_{j}$ must be positive semi-definite. As the covariance is small in large systems, we can approximate *μ*_*g*_ neglecting the covariance term.

Similar to the mean *μ*_*g*_, we can approximate the variance ${\sigma }_{g}^{2}$. For ${\sigma }_{g}^{2}$, however, bounding the covariance terms is more challenging and requires further approximations (see Supplementary Appendix D), which we verified numerically (Supplementary Appendix E). We obtain the following equation for the variance:

10 $${\sigma }_{g}^{2}={\sigma }_{r}^{2}+(n-1)cP(({\mu }_{x}^{2}+{\sigma }_{x}^{2})({\mu }_{a}^{2}+{\sigma }_{a}^{2})-cP{\mu }_{x}{\mu }_{a}^{2})+n\zeta .$$

Thereby, *ζ* represents the unknown covariance terms, which are of constant order of magnitude. Numerical estimates suggest that $\zeta \ge 0$. Though we could show that the variance ${\sigma }_{g}^{2}$ is not dominated by covariance terms, *ζ* may still have a significant impact.

**Figure 1:**
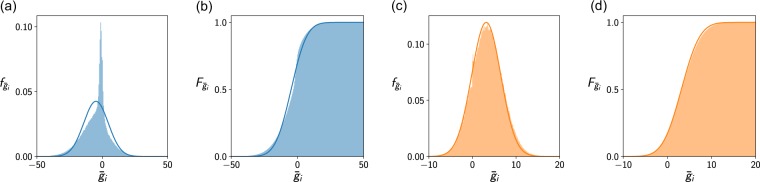
Probability density function ${f}_{{\bar{g}}_{i}}$ (**a**,**c**) and cumulative density function ${F}_{{\bar{g}}_{i}}$ (**b**,**d**) of the distribution of ${\bar{g}}_{i}$ for a system with few persisting species (**a**,**b**) and many persisting species (**c**,**d**). The shaded areas correspond to histograms of the ${\bar{g}}_{i}$ values obtained in simulations. The solid lines depict our approximation of the distributions. In the system with few coexisting, our approximation of the cumulative density function is precise (**b**) even though the observed probability density function differs significantly from our approximation (**a**). In the case with many persisting species, the observed and approximate distribution are very close. Refer also to Supplementary Appendix F for details on the method used to generate the figures.

After computing the mean and the variance of ${\bar{g}}_{i}$, we can attempt to approximate the cumulative density function of ${\bar{g}}_{i}$, which we need to determine ${\mathbb{P}}({\bar{g}}_{i}\ge 0)$. Recall that ${\bar{g}}_{i}={r}_{i}+{\sum }_{j\in {I}_{i}}{a}_{ji}{\bar{x}}_{j}$. If the summands ${a}_{ji}{\bar{x}}_{j}$ were independent of each other, the sum ${\sum }_{j\in {I}_{i}}{a}_{ji}{\bar{x}}_{j}$ would be approximately normally distributed according to the central limit theorem. However, if the summands are positively correlated, their sum will take both more extreme values and more values close to zero (see Fig. 1a).

The frequency of extreme values has a large effect on the variance of a random variable. We have already noted that the covariance terms do not change the order of magnitude of the variance ${\sigma }_{g}^{2}$. Consequently, the weight that the correlations add on the tails of the distribution is relatively small. Thus, we may ignore these correlations when we approximate the cumulative density function of ${\bar{g}}_{i}$ (see Fig. 1).

#### **Approximation 1**.

*The average non-truncated growth rate is normally distributed with mean μ*_g_ *and variance* ${\sigma }_{g}^{2}$,

11 $${\bar{g}}_{i}={r}_{i}+\sum _{j\in {I}_{i}}\,{a}_{ji}{\bar{x}}_{j}\mathop{\approx }\limits^{d}{\rm{N}}({\mu }_{g},{\sigma }_{g}^{2}),$$

*whereby the covariance terms in μ*_*g*_ *and* ${\sigma }_{g}^{2}$ *are ignored*.

Our expressions for *μ*_*g*_ and ${\sigma }_{g}^{2}$ contain the unknown mean *μ*_*x*_ and variance ${\sigma }_{x}^{2}$ of the average density of persisting species. In general, it is difficult to find rigorous approximations of *μ*_*x*_ and ${\sigma }_{x}^{2}$, as they strongly depend on how many species reach the limiting density. Nonetheless, it is possible to obtain reasonable estimates for the quantities as we will see below.

If many species grow up to their capacities, they will have either a very high or a moderate average density. Therefore, the variance will be large. Since the population densities are constrained to be in the interval $(0,1]$, the variance is bounded by ${\sigma }_{x}^{2}\le {\mu }_{x}(1-{\mu }_{x})$ and we may approximate ${\sigma }_{x}^{2}\approx {\omega }_{1}{\mu }_{x}(1-{\mu }_{x})$ with some factor ${\omega }_{1}$. Choosing ${\omega }_{1}=1$ would be equivalent to assuming that the average densities are Bernoulli distributed. Following numerical observations (see Supplementary Appendix E), we set ${\omega }_{1}=\frac{1}{2}$.

If most species densities are bounded, it is reasonable to assume that the average densities of surviving species are approximately uniformly distributed around their mean *μ*_*x*_. With this assumption, we arrive at ${\sigma }_{x}^{2}\approx {\omega }_{2}\frac{{\mu }_{x}^{2}}{3}$, whereby ${\omega }_{2}$ is again a scaling factor. A choice of ${\omega }_{2}=1$ is equivalent to assuming that the average densities follow a uniform distribution. In simulations ${\omega }_{2}=2$ appeared to be a good choice (Supplementary Appendix E). Either way, the factors ${\omega }_{1}$ and ${\omega }_{2}$ have only minor effects on the results. We summarize our approximation below.

#### **Approximation 2**.

*If many surviving species grow to their capacity*, *it is* ${\sigma }_{x}^{2}\approx \frac{1}{2}{\mu }_{x}(1-{\mu }_{x})$, *otherwise* ${\sigma }_{x}^{2}\approx \frac{2}{3}{\mu }_{x}^{2}$.

To derive an estimate of *μ*_*x*_ and to find out whether species are likely to reach their limiting densities, let us consider a species *i* that is known to never hit its limiting density. Then it must hold

12 $$0={\bar{g}}_{i}-{a}_{ii}{\bar{x}}_{i}.$$

Applying conditional expectation yields

13 $$0={\mathbb{E}}({\bar{g}}_{i}-{a}_{ii}{\bar{x}}_{i}|{\bar{x}}_{i} > 0)={\mathbb{E}}({\bar{g}}_{i}|{\bar{g}}_{i} > 0)-{\mathbb{E}}({a}_{ii}{\bar{x}}_{i}|{\bar{x}}_{i} > 0),$$

whereby we used that ${\bar{x}}_{i} > 0$ is equivalent to ${\bar{g}}_{i} > 0$. For each given *P* and *μ*_*x*_, the distribution of ${\bar{g}}_{i}$ can be estimated according to Approximation 1, which implies that ${\mathbb{E}}({\bar{g}}_{i}|{\bar{g}}_{i} > 0)$ is the mean value of a truncated normal distribution. If *a*_*ii*_ is chosen deterministically or with small variance, we can furthermore approximate ${\mathbb{E}}({a}_{ii}{\bar{x}}_{i}|{\bar{x}}_{i} > 0)\approx {\mathbb{E}}({a}_{ii}){\mu }_{x}$. This in combination with Approximation 2 allows us to solve Eq. (13) numerically for *μ*_*x*_ for each given *P*.

It can happen that Eq. (13) does not have a solution in the admissible interval $(0,1]$. This indicates that the species hit their density bounds. In this case there is little we can know about *μ*_*x*_ other than that it is relatively large. Therefore, we may consider different large values of *μ*_*x*_ to assure that our results are not sensitive to an arbitrary choice of *μ*_*x*_. Numerical simulations (Supplementary Appendix E) also support choosing a large value of *μ*_*x*_ (e.g. ${\mu }_{x}=0.8$).

Applying the findings and approximations above, we can write ${\mu }_{g}(P)$ and ${\sigma }_{g}^{2}(P)$ as functions of the expected fraction *P* of coexisting species. With (6) and (11), we arrive at an implicit equation,

14 $$P={S}_{{\rm{N}}}(0;{\mu }_{g}(P),{\sigma }_{g}^{2}(P)),$$

which can be solved for *P* numerically. Here, *S*_N_ is the survival function (complementary cumulative density function) of the normal distribution, which results from integrating over the positive branch of the probability density function of ${\bar{g}}_{i}$. The functions ${\mu }_{g}(P)$ and ${\sigma }_{g}^{2}(P)$ are computed according to Eqs. (7) and (10), respectively.

### Refined heuristic

In the previous section, we derived a heuristic for the expected fraction *P* of species coexisting in large and dense systems. In these networks, it is likely that every species is affected by some other species. In sparse networks, however, some species may *not* be affected by *any* other species. Survival of such isolated species does not depend on the distribution of the interaction constants *a*_*ji*_. Below, we derive a refined model that accounts for isolated species.

If a species *i* is not affected by any other surviving species, then it survives if and only if it is a primary producer:

15 $$\begin{array}{rcl}{\mathbb{P}}(i\,{\rm{survives}}|{\rm{not}}\,{\rm{affected}}) & = & {\mathbb{P}}({r}_{i} > 0)\\  & = & {S}_{{\rm{N}}}(0;{\mu }_{r},{\sigma }_{r}^{2}).\end{array}$$

On the other hand, if species *i* is affected by some surviving species, then *i*’s survival is given by the sign of the per-capita growth rate

16 $${\mathbb{P}}(i\,{\rm{survives}}|{\rm{affected}})={\mathbb{P}}({r}_{i}+\sum _{j\in {I}_{i}}\,{a}_{ji}{\bar{x}}_{j}\ge 0|\exists j\in {I}_{i}:{a}_{ji}{\bar{x}}_{j}\ne 0).$$

The right hand side of Eq. (16) is well approximated by the probability that we have estimated in the previous section.

#### **Approximation 3**.

$${\mathbb{P}}(i\,survives|affected)\approx {S}_{{\rm{N}}}(0;{\mu }_{g}({n}_{P}),{\sigma }_{g}^{2}({n}_{P}))$$.

To derive the probability that species *i* is affected by some other species, we consider a network in which *n*_*P*_ species coexist. Recall that *c* is the probability that a link exists, i.e. $c={\mathbb{P}}({a}_{ji}\ne 0)$. Hence, the probability that a species *i* is affected by some other persisting species is approximately

17 $$\begin{array}{rcl}{\mathbb{P}}({\rm{affected}}|{n}_{P}\,{\rm{species}}\,{\rm{survive}}) & \approx  & 1-{(1-c)}^{{n}_{P}}\\  & \approx  & 1-\exp (\,-\,c{n}_{P})\end{array}$$

and

18 $$\begin{array}{rcl}{\mathbb{P}}({\rm{affected}}) & \approx  & {\mathbb{E}}(1-\exp (\,-\,c{n}_{P}))\end{array}$$

19 $$\,\le \,1-\exp (\,-\,cnP)$$

by Jensen’s inequality. As we do not know the distribution of $\exp (-c{n}_{P})$, we apply a first-order Taylor expansion to estimate the expected value on the right hand side of (18) and arrive at the right hand side of (19).

#### **Approximation 4**.

$${\mathbb{P}}(affected)\approx {\mathbb{E}}(1-\exp (\,-\,c{n}_{P}))\approx 1-\exp (\,-\,cnP)$$.

This approximation is imprecise, if *cn* is large and *P* is small. Though it is possible to correct for the error introduced by this approximation, we refrain from making our model more complicated here. Instead we note that our heuristic will provide upper or lower bounds on *P* dependent on whether ${\mathbb{P}}(i\,{\rm{survives}}|{\rm{affected}}) > {\mathbb{P}}(i\,{\rm{survives}}|{\rm{not}}\,{\rm{affected}})$ or not, respectively.

Putting the pieces together, we obtain

20 $$\begin{array}{rcl}P & = & {\mathbb{P}}({\rm{affected}}){\mathbb{P}}(i\,{\rm{survives}}|{\rm{affected}})\,+\,(1-{\mathbb{P}}({\rm{affected}})){\mathbb{P}}(i\,{\rm{survives}}|{\rm{not}}\,{\rm{affected}})\\  & = & {\mathbb{P}}(i\,{\rm{survives}}|{\rm{affected}})\,+\,{\mathbb{P}}({\rm{not}}\,{\rm{affected}})({\mathbb{P}}(i\,{\rm{survives}}|{\rm{not}}\,{\rm{affected}})-\,{\mathbb{P}}(i\,{\rm{survives}}|{\rm{affected}}))\end{array}$$

21 $$\approx \,{S}_{{\rm{N}}}(0;{\mu }_{g}(P),{\sigma }_{g}^{2}(P))+\exp (-cnP)({S}_{{\rm{N}}}(0;{\mu }_{r},{\sigma }_{r}^{2})-\,{S}_{{\rm{N}}}(0;{\mu }_{g}(P),{\sigma }_{g}^{2}(P))).\,$$

**Figure 2:**
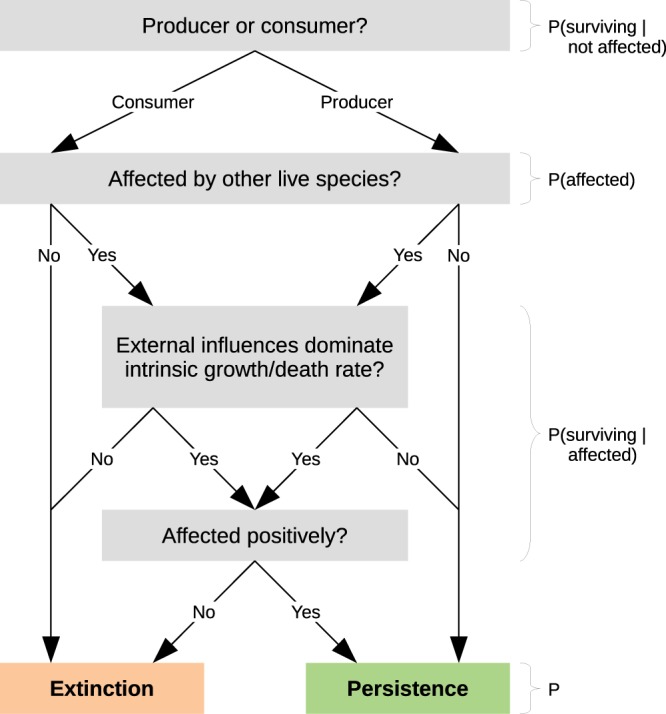
Flow chart for the refined persistence heuristic. To persist, a species must either be a primary producer without strong predators or competitors, or the species must be a consumer with strong mutualistic partners or prey in sufficient abundance. The probability *P* that a species persists can be determined by computing the products of the corresponding probabilities along the paths that lead to persistence.

The solutions of this equation constitute our heuristic persistence estimates. Figure 2 depicts the rationale behind Eq. (20) as a flow chart.

### Simulation method

We briefly outline the simulation approach that we used to verify the heuristic estimates. We applied Monte Carlo simulations to determine how many species are expected to persist in the randomly assembled networks. We examined networks varying in the following properties: (1) species richness *n* (2) connectivity *c*, (3) mean *μ*_*r*_ and variance ${\sigma }_{r}^{2}$ of the growth rates *r*_*i*_, (4) mean *μ*_*a*_ and variance ${\sigma }_{a}^{2}$ of the interaction parameters *a*_*ji*_, (5) the fraction *b* of initial high-density species.

For each set of network properties, we randomly generated 1000 networks and solved the respective dynamical system (2) for $T=1000$ time steps with a fourth order Runge-Kutta method with step size 0.01. For each network, we noted which proportion of the species had an average density larger than $\varepsilon =0.001$ during the last quarter of the simulated time interval; i.e. in $[\tfrac{3}{4}T,T]$. We took the average of these proportions for all 1000 networks and obtained by this means an estimate of the expected number *P* of coexisting species.

**Figure 3:**
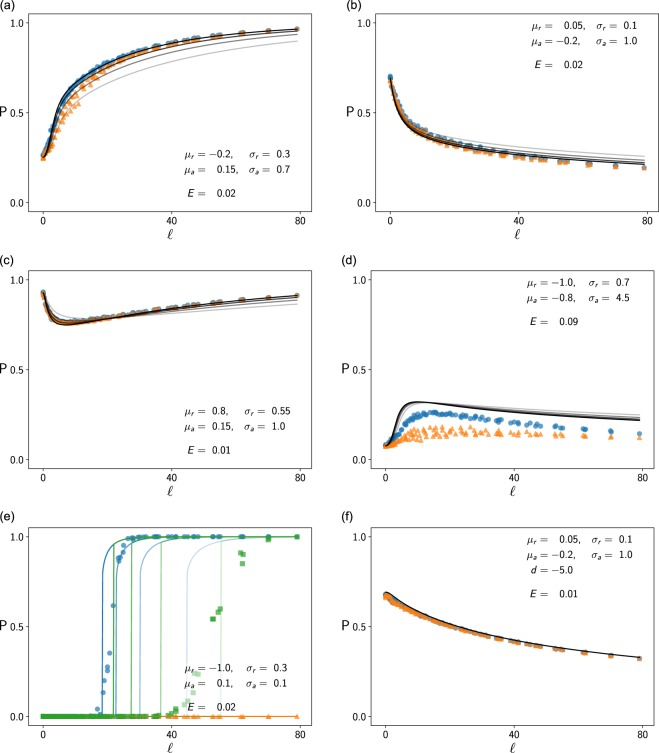
Proportion *P* of coexistent species as function of the linkage density *l*. Each subfigure corresponds to networks with different distributions of growth and interaction constants, respectively. Each marker represents a network with specific species richness *n* and connectivity *c*. The blue dots refer to networks with high initial species densities ($b=1$), and orange triangles to networks with very low initial densities (colonization scenario, $b=0$). The green squares in (**e**) depict an intermediate scenario with $b=0.4$. The solid lines show our heuristic estimates. In all subfigures except for (**f**), the species densities hit the density bound, and the variable *μ*_*x*_ cannot be determined exactly. In these cases, multiple lines depict the heuristic estimates for different values of *μ*_*x*_. The opacity of the lines corresponds to the values for *μ*_*x*_ ranging from 0.4 to 1 (high values correspond to low transparency). It is visible that predictions with high values of ${\mu }_{x}$ meet our observations best, in general. The number *E* denotes the mean error between observations and predictions (${\mu }_{x}=0.8$ in all subfigures except (**f**)). In (**e**), we computed the error for the scenario $b=1$ only.

This approach is computationally costly. Hence, we focused on few combinations of the parameters *μ*_*r*_, ${\sigma }_{r}^{2}$, *μ*_*a*_, and ${\sigma }_{a}^{2}$ (given in Fig. 3). For each parameter tuple $\Theta =({\mu }_{r},{\sigma }_{r}^{2},{\mu }_{a},{\sigma }_{a}^{2})$, we simulated networks with many combinations of species richness *n* and connectivity *c*. We considered 10 different values for *n* and *c*, respectively. Thereby, we let *n* range from 4 to 80 and *c* range from 0.001 to 1 in approximately equidistant steps. We performed a Monte Carlo simulation for each of the 100 combinations of *n* and *c*.

To test how the results depend on the initial condition, we examined the networks in a colonization scenario (*b* = 0) and in a scenario with high initial densities (*b* = 1), respectively. Furthermore, we considered different choices for the intra-specific competition terms *a*_*ii*_. In most of our simulations, we drew the parameters *a*_*ii*_ from a normal distribution with variance ${\sigma }_{a}^{2}$ centred and truncated at 0. However, to test the validity of our heuristic in scenarios where species do not hit the sharp density bound, we also simulated systems with specific, strongly negative values ${a}_{ii}=d$.

To validate our model in the broadest-possible range of scenarios, we tested $\Theta$-tuples for which our heuristic predicted qualitatively different results. Furthermore, we verified that our simulation time horizon *T* was sufficiently large by extending *T* to 2000. Finally, we tested how our results would change if we exchanged the normal distribution for the parameters *r*_*i*_ and *a*_*ji*_ with a uniform distribution with similar mean and variance. In total, we simulated and analysed more than 2 million networks.

## Results

### Theoretical results

The heuristic we derived provides us with estimates of how many species coexist in a system with given properties. In this section, we analyse our heuristic (21) and describe how the different network characteristics affect persistence. A summary of the possible qualitative complexity-persistence relationships can be found in Table 2.

**Table 2:** Possible qualitative complexity-persistence relationships.

Complexity-Persistence Relation	Condition	Description
Monotonously increasing	${\mathbb{P}}({r}_{i} > 0)\le {\mathbb{P}}({a}_{ji} > 0)\ge \frac{1}{2}$	Persistence increases with the linkage density. Thereby, *P* converges to 1 if ${\mu }_{a} > 0$ and to $\frac{1}{2}$ if ${\mu }_{a}=0$.
Monotonously decreasing	${\mathbb{P}}({r}_{i} > 0)\ge {\mathbb{P}}({a}_{ji} > 0)\le \frac{1}{2}$	Persistence decreases with the linkage density. Thereby, *P* converges to 0 if ${\mu }_{a} < 0$ and to $\frac{1}{2}$ if ${\mu }_{a}=0$.
Constant	${\mathbb{P}}({r}_{i} > 0)={\mathbb{P}}({a}_{ji} > 0)=\frac{1}{2}$	Half of the species persist regardless of the linkage density.
Local persistence minimum	${\mathbb{P}}({r}_{i} > 0) > {\mathbb{P}}({a}_{ji} > 0) > \frac{1}{2}$	For networks with small linkage density, addition of links has negative effects on persistence. If the linkage density is large, persistence increases with the linkage density and *P* approaches 1.
Local persistence maximum	${\mathbb{P}}({r}_{i} > 0) < {\mathbb{P}}({a}_{ji} > 0) < \frac{1}{2}$	For networks with small linkage density, addition of links has positive effects on persistence. If the linkage density is large, persistence decreases with the linkage density and *P* approaches 0.
Persistence bistability	Eq. (21) has multiple solutions for networks with high linkage densities; this requires ${\mathbb{P}}({r}_{i} > 0) < {\mathbb{P}}({a}_{ji} > 0)$, ${\mathbb{E}}(|{r}_{i}|)\gg {\mathbb{E}}(|{a}_{ji}|)$. Alternatively: Eq. (13) has one solution at low linkage densities and no solution at high linkage densities; this requires ${\mathbb{E}}({a}_{ii})\ll 0$.	In networks with small linkage density, few species persist. If the linkage density is sufficiently large, *P* can take a large or a small value, dependent on the initial condition. The system exhibits points of no return and the removal of species can result in system collapse. The higher the linkage density the greater the probability that the larger persistence value is attained. Bistability can be suppressed by choosing strongly negative intra-specific competition constants *a*_*ii*_, which however induce bistability at high linkage densities.

We start by considering the species richness *n* and the connectivity *c*. With *n* and *c*, we can compute the expected number of links per species $l:=(n-1)\cdot c$, which is called the *linkage density*. The right hand side of Eq. (21) can be written in terms of the linkage density *l* and the connectivity *c*. It can be shown that *c* has only a marginal effect on *P* under the new parameterization. This implies that persistence is mainly driven by the linkage density *l* and not by size or the connectivity of the network.

To understand how the linkage density affects persistence, we consider the limit cases with small and large linkage densities. If no links exist, i.e. $l=0$, Eq. (21) turns into an explicit equation in which *P* is equal to the expected number of producers. As more links are added, the proportion of producers becomes irrelevant and persistence depends only on the distribution of the interaction constants *a*_*ji*_. In the limit of many links per species, i.e. $l\to \infty$, all species survive if ${\mu }_{a} > \frac{1}{2}$, and all species go extinct if ${\mu }_{a} < \frac{1}{2}$ (see examples in Fig. 3). Only if ${\mu }_{a}=\frac{1}{2}$, the variance (10) becomes much larger than the mean (7) and half of all species persist.

For intermediate linkage densities, the heuristic predicts a wide range of possible dependencies of *P* on *l*. If the linkage density is small, *P* increases with *l* if the the probability of a positive link is larger than the probability of a positive growth rate, i.e. ${\mathbb{P}}({a}_{ji} > 0) > {\mathbb{P}}({r}_{i} > 0)$. In the opposite case, *P* decreases with *l*. This criterion holds independently of whether the links are positively or negatively biased on average. Hence, local persistence minima and maxima are possible (see examples in Fig. 3c,d). Since persistence increases at small linkage densities if few primary producers are present, whereas negatively biased links decrease persistence at high linkage densities, $\frac{1}{2} > {\mathbb{P}}({a}_{ji} > 0) > {\mathbb{P}}({r}_{i} > 0)$ implies that *P* has a local maximum at intermediate linkage densities. With similar reasoning we find that *P* has a local minimum if ${\mathbb{P}}({r}_{i} > 0) > {\mathbb{P}}({a}_{ji} > 0) > \frac{1}{2}$.

The curvature of $P(l)$ depends on the distribution of the intrinsic growth/death rates *r*_*i*_ and how quickly they are dominated by the interaction terms. For example, if the intrinsic growth rates have a strong negative bias, i.e. ${\mu }_{r}\ll 0$ and ${\sigma }_{r}^{2}$ is small, then a small number of interactions does not suffice to keep species alive. However, the chances of survival increase rapidly once there are sufficiently many links to overcome the negative growth bias. Therefore, the right hand side of our persistence heuristic (21) increases strongly sigmoidly with *l* in these cases.

**Figure 4:**
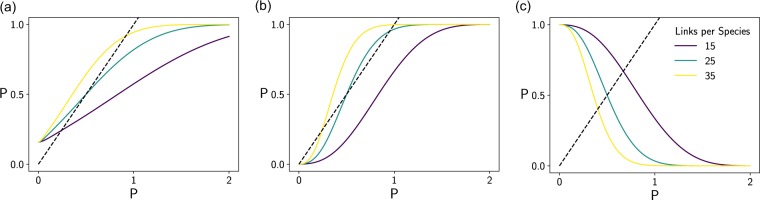
Left hand side (dashed black line) and right hand side (solid coloured lines) of Eq. (21) as functions of *P*. Each subfigure represents systems with specific distributions of the model parameters, respectively. The three coloured lines in each subfigure stand for networks differing in linkage density *l*: purple: *l* = 15; green: *l* = 25; yellow: *l* = 35. Our persistence estimates are the points where the solid lines intersect with the dashed line, respectively. The systems in (**a**,**c**) have unique persistence estimates. For the systems depicted by the green and yellow curve in (**b**), we obtain multiple possible persistence estimates. Note that if the solid lines are decreasing (**c**), the persistence estimate is always unique. The horizontal axis includes impermissibly high values of *P* to better depict the general shape of the curves. (Parameters: (**a**) ${\mu }_{r}=-\,1$, ${\sigma }_{r}^{2}=1$, ${\mu }_{a}=0.1$, ${\sigma }_{a}^{2}=0.01$; (**b**) ${\mu }_{r}=-\,1$, ${\sigma }_{r}^{2}=0.1$, ${\mu }_{a}=0.1$, ${\sigma }_{a}^{2}=0.01$; (**c**) ${\mu }_{r}=1$, ${\sigma }_{r}^{2}=0.1$, ${\mu }_{a}=-\,0.1$, ${\sigma }_{a}^{2}=0.01$. The remaining parameters are similar in all Figures: $c=0.5$, ${\mu }_{x}=0.8$).

The shape of the right hand side of Eq. (21) has a strong influence on the system dynamics (see Fig. 4). If the function increases sigmoidly, Eq. (21) can have up to three solutions, one of which must be repelling (see Fig. 4b). In these cases, the number of persisting species depends on the initial condition.

We may call parameter combinations at which Eq. (21) has exactly two solutions bifurcation points. At these points in the parameter space, the number of solutions of (21) transitions between one and three (see Fig. 4b). Consequently, small alterations of the system can have a strong effect: removal of a few links can lead to an almost complete collapse of the system, whereas addition of links can give rise to a large system that would break down otherwise (see example in Fig. 3e). Note that this bistable system behaviour can only occur in systems with many consumers heavily reliant on other species. According to our heuristic, systems with many primary producers will never be bistable (see Fig. 4c). That is, addition of a link may have detrimental, but not fatal consequences on average.

While the distributions of the parameters *r*_*i*_ and *a*_*ji*_ play a major role in the persistence heuristic, the value of the sharp capacity *K* and the intra-specific competition constants *a*_*ii*_ effect persistence only through the average densities of surviving species. If the *a*_*ii*_ are not strong enough to bound species densities, the effect of these parameters can be neglected. Instead, the capacities *K*_*i*_ govern the mean average density *μ*_*x*_ of surviving species. Hence, choosing different values for the capacities *K*_*i*_, possibly at random, would only require us to adjust our constant estimate for *μ*_*x*_. This is easy to do and does not affect the qualitative system dynamics.

Only if intra-specific competition is sufficiently large to bound the species densities, the *a*_*ii*_ affect the average density of surviving species *μ*_*x*_. In that event, stronger competition leads to smaller average densities, and interactions between species have a smaller weight compared to intrinsic growth and death. Consequently, persistence does not change as rapidly with the linkage density, and bistable behaviour is suppressed^21^ (see example in Fig. 3f). However, strong competition can also induce bistable behaviour when the self-damping is overruled by positive interactions, and the Eq. (13) for *μ*_*x*_ has two zeros.

### Simulation results

In the previous section, we have outlined the persistence predictions of our heuristic. In this section, we present simulation results that allow us to assess the precision of the predictions.

In most instances, the simulation results matched our heuristic predictions well (see Fig. 3). Especially in networks with high persistence predictions, the heuristic was very precise (Fig. 3a–c,f). In networks with small predicted *P*, our heuristic correctly predicted the order of magnitude of *P* and the shape of the persistence curve $P(l)$. However, the predicted persistence values deviated from the observed values by up to 0.2 (Fig. 3d).

In networks for which we obtained a unique persistence estimate, the fraction *b* of initial native species had a small effect on the results. In colonization scenarios ($b=0$), simulated persistence was slightly lower. As suggested by the heuristic, this was different in “bistable persistent” networks, that is, networks for which our persistence model (21) has multiple solutions. Despite predicting the qualitative behaviour correctly, the heuristic failed to predict precisely at which linkage density the community would collapse or allow colonization if $b < 1$. In addition, the simulations showed a less sharp threshold than estimated.

Extending the time horizon *T* did not have a notable effect in our simulations except with bistable networks, in which the ascend at the threshold value became sharper with a longer time horizon. Similarly, simulations in which the parameters were drawn form a uniform distribution did not differ from simulations using normal distributions with equal means and variances.

## Discussion

We introduced a novel heuristic approach to estimate the fraction of persisting species in complex dynamical systems. Applying the method to a model of ecological networks, we showed that the heuristic can provide valuable ecological insights. Similar to earlier studies, our results suggest that the linkage density is a major driver of stability or persistence^2,7,25,26^. However, we did not observe a generally applicable complexity-stability relationship. Instead, an increase of species richness and connectivity favoured or hindered persistence depending on the fraction of primary producers and the distribution of the interaction parameters.

While it has been shown earlier that the impact of complexity on persistence depends on the sign of the mean interaction strength^16^, our study adds on to earlier results by also identifying more subtle system behaviour. For example, even if a high linkage density will result in the majority of the species going extinct, a small number of links can have a positive effect on persistence. Introduction of a moderate number of species with a negative average impact can increase persistence if these species serve as resources for some species that would go extinct otherwise, e.g. due to lack of food. Similarly, introduction of a small number of species from which most other species benefit can decrease persistence if the species in the original network would have persisted anyway.

An interesting phenomenon we observed is persistence bistability, i.e. the existence of systems in which persistence depends on the initial species densities. Thereby, persistence bistability refers to the *number* or *fraction* of persisting species, not their *density* or *identity*. A system can be multistable and still have a unique set of persisting species. Similarly, multistable systems may have distinct, but on average equally sized sets of persisting species. In a system exhibiting *persistence bistability*, however, different *numbers* of species will persist depending on the initial conditions. Hence, persistence bistability is a special case of multistability.

Species in systems exhibiting persistence bistability heavily rely on multiple other species, and if one of these species goes extinct, this triggers a “snowball-effect”, a cascade of extinctions. Conversely, such networks cannot establish, unless all the required species are present in sufficient density. This can make it difficult to restore these systems after disturbances. The behaviour of persistence bistable systems may be understood as bootstrap percolation^27,28^, modelling systems in which ‘sites’ are ‘activated’, if enough neighbouring sites are active.

As bistable systems exhibit “points of no return”, identifying such systems can be of high importance in conservation and restoration ecology^29–31^. For our model, our heuristic allows us to characterize systems with persistence bistability precisely, and some identified principles may even hold with higher generality. For example, persistence bistability requires that most of the species in the network are consumers. In the opposite case, in which the network consists mostly of producers, introduction of species may have detrimental but not devastating effects. We suggest that it would be worthwhile to grant higher attention to the phenomenon of persistence bistability in the complexity-stability debate, and our study provides a tool to analyse systems with this behaviour.

### Application in invasion ecology: biotic resistance and invasional meltdown hypothesis

Our results suggest that the probability that a species survives in a random system is barely affected by its initial density, unless the systems exhibit persistence bistability. Therefore, our heuristic can be applied to scenarios in which species invade pre-existing ecosystems. Hence, our study may contribute to the invasion ecology debate on the impact of primary invasions on the success of secondary invasions^32^. The biotic resistance hypothesis^33–35^ suggests that introduction of a primary invasive species decreases the likelihood of successful secondary invasions. In contrast, the invasional meltdown hypothesis^36^ states that introduction of invasive species increases the vulnerability of the system and therefore facilitates secondary invasions. Both competing theories have been supported and challenged by empirical findings^32,37^.

According to our heuristic, primary invaders can either improve or decrease the chances of secondary invasions, dependent on how the primary invaders affect the linkage density and the distribution of interaction strengths. Note, however, that our model does not distinguish between successful and unsuccessful primary invaders. That is, even if a primary invader is unsuccessful and has a small but positive density, it may affect the success of future invasions. To study the impact of successful primary invaders only, the respective conditional probability for persistence must be found. This is a task for future research.

### The heuristic approach

We obtained the results presented above by applying a novel heuristic approach. Compared to analytical methods, the heuristic approach is less rigorous and leaves more room for inaccuracies. Nonetheless, our heuristic results matched numerical results remarkably well, and the resulting equation for persistence allows an in-depth mathematical analysis with reasonable effort. This, in turn, can increase our understanding of the general mechanisms behind persistence. The basic idea of considering time-averages to study extinction of species in dynamical systems is well established^21,38^.

Though other analytical techniques used to study persistence may yield similar insights as our approach and be more precise, our method has the advantage that it permits the analysis of systems that are not guaranteed to have a unique set of persisting species. This increase in generality has considerable advantages. First, it allows us to study systems with more complicated model behaviour, such as systems with persistence bistability. Second, the wide range of permissible model parameters makes it possible to compare purely competitive or mutualistic systems to predator-prey or unrestricted systems. Thus, the heuristic approach may have the potential to synthesize earlier theoretical results obtained for distinct classes of systems^3^.

Our approach allowed us also to reproduce and generalize some earlier findings on diagonally stable systems. These systems are known to have a unique globally attractive equilibrium^21^. For such systems, the fraction of persisting species has been shown to be 1/2 independent of the size and connectivity of networks as long as the intrinsic growth rates *r*_*i*_ and the interaction parameters *a*_*ji*_ are sampled from distributions symmetric around 0^5^. This agrees with our heuristic estimate. Furthermore, persistence has been studied in competitive networks with random growth constants *r*_*i*_ and deterministic competition parameters *a*_*ji*_^5^. Our heuristic adds on to these results by predicting how persistence changes if the *a*_*ji*_ are chosen at random.

### Sources of inaccuracies

Our heuristic estimates for the fraction of coexisting species agree well with the results obtained in numerical simulations. This indicates that our simplifications do not have a strong effect on the results. The existence assumption for the mean species densities is known to hold in many systems of interest, including systems in which the sharp threshold is not hit^21^ and systems approaching some kind of steady state or limit cycle. We have not observed any indication that the assumption undermines the applicability of our heuristic.

Nonetheless, some considerable inaccuracies occurred in large systems with a dominance of negative links and therefore small fraction of persisting species. A closer investigation of these systems showed that the errors are due to the poor approximation of the mean of the exponential term in our heuristic (Approximation 4). This weakness can be corrected for with additional approximations.

Large quantitative deviances between the heuristic estimates and the simulation results occurred only when we attempted to find the tipping point linkage densities in bistable systems. The exact initial densities of the species are relevant in these scenarios. As our heuristic is based on the long-time behaviour of the system, it does not provide a clear relationship between initial conditions and persistence. Nevertheless, the heuristic correctly identified the parameter ranges that lead to persistence bistability.

### Ecological limitations and possible extensions

We considered a class of rather abstract ecosystem models in this paper. Thus, the studied systems might lack features found in natural systems, and simulation approaches^7,13,14^ may remain the tool of choice to analyse models with strong focus on realism. Nonetheless, we argue that many of our model’s limitations either have a minor effect on the results or can be accounted for by adjusting the heuristic to fit more intricate models.

First, our model does not bound the strengths of species interactions with functional response terms. Instead, we inhibited unbounded species growth by introducing a simple density bound without a clear mechanistic justification. However, our heuristic can be directly applied to a slightly adjusted model with bounded interactions instead of a sharp density bound (see Supplementary Appendix G). Therefore, we hypothesize that introduction of functional response will only affect the quantitative but not the qualitative results of this study. Nevertheless, it would be desirable and, we believe, possible to adjust our heuristic to study models with functional response. Despite this potential for improvement, our heuristic can be directly applied to Lotka-Volterra systems that are stable by construction. Thus, our work adds on to earlier studies of these systems^5,16^.

Second, the interaction constants are unlikely to be independent in real ecosystems, and predator-prey dependencies may occur with a different frequency than mutualistic or competitive interactions^26,39,40^. However, we conjecture that introduction of correlations between interaction terms will not undermine the applicability of our heuristic. This would go in line with earlier results finding that correlations between interaction parameters do not change persistence in systems with zero-mean interaction distributions^5^. The task of future research would be to adjust our covariance bounds to confirm that the computed expected values are not significantly affected by these terms.

Third, the topological structure of natural systems is rarely random as assumed here^41,42^, since trophic, mutualistic, and competitive interactions impose a certain structure on networks^43,44^ and the network structure is dependent on the ecological processes behind the network assembly^20^. This limitation may be the hardest to address, because our heuristic exploits an exchangeability property often not found in systems with a more realistic structure. Since the network structure affects stability^8,13,14,39,45^, it will be a worthwhile task for future research to apply our heuristic approach to models with a more realistic network structure. A first step in this direction is to split the network into multiple similar subnetworks. As this model modification does not affect the exchangeability assumption, our heuristic can be almost directly applied to bipartite networks with similar species groups. A further step would be to study networks with different classes of species. The result would be a heuristic consisting of a system of equations. Though such an equation system may be harder to analyse than the simple one-dimensional heuristic derived in this paper, it may still be possible to gain significant insights with this approach.

## Conclusion

The main result of this paper is that a simple heuristic suffices to predict important aspects of the complex behaviour of large dynamical systems. The derivation of our heuristic is based on approximations, but the precision of our estimates throughout our simulations is remarkable. Our heuristic predicted the correct qualitative system behaviour in all simulations we conducted.

The heuristic approach allows the study of persistence in ecological networks without strong restrictions on model parameters. Thus, the method is suitable to compare different classes of ecosystems and to investigate phenomena that were hard to analyse with classical techniques. We hope therefore that future studies will build on our observations and apply our method to more sophisticated models. The results could synthesize earlier findings on ecosystem stability and may be a step forward to a general framework of complexity and stability.

## Supplementary information

Supplementary Appendices

## Data Availability

No datasets were generated or analysed during the current study.
