# Supplementary material for: An Approach to Study Species Persistence in Unconstrained Random Networks
Source: Sci Rep. 2019 Oct 1;9:14110. doi: 10.1038/s41598-019-50373-z (PMC6773691; doi:10.1038/s41598-019-50373-z)
Supplement: Supplementary file 1 — Supplementary Appendices [file 41598_2019_50373_MOESM1_ESM.pdf]

Supplementary Appendices for ‘An Approach to Study Species Persistence in Unconstrained Random Networks’

Samuel M. Fischer and Andreas Huth

A Existence and uniqueness of solutions

In this appendix, we show that a unique solution exists for the initial value problem $\mathbf{x}(0) = \mathbf{x}_0 \in [0,1]^n$,

$$\frac{dx_i}{dt} = \begin{cases} x_i g_i(\mathbf{x}) & \text{if } x_i < 1 \text{ or } g_i(\mathbf{x}) < 0 \\ 0 & \text{else,} \end{cases} \tag{S1}$$

with $i \in I := \{1, \ldots, n\}$, $\mathbf{x} = (x_1, \ldots, x_n)$, $g_i(\mathbf{x}) := r_i + \sum_{j \in I} a_{ji} x_j$. Note that unlike in the main text, g_i defined here includes the term $a_{ii} x_i$.

It is easy to extend the presented results also for other initial conditions (with entries larger than 1) and for capacities $K_i \neq 1$ (refer to system (2) in the main text).

A.1 Existence of solutions

As the right hand side of system (S1) is discontinuous, there will be be conditions at which the solution to (S1) is not differentiable.Therefore, we need a sufficiently general notion of solution of the differential equation. We say [1] that $\mathbf{x}$ is a solution to system (S1) if (S1) is satisfied almost everywhere and $\mathbf{x}$ is absolutely continuous. This is equivalent to claiming that $\mathbf{x}$ solves the integral version of (S1) [1]. Such solutions are called Caratheodory solutions [2].

An introduction to differential equations with discontinuous right hand sides is given in [1]. Refer to reference [2] for a more in-depth analysis of differential equations with discontinuous right hand sides.

Lemma 1. *System (S1) has a solution for any initial condition* $\mathbf{x}(0) = \mathbf{x}_0 \in [0,1]^n$.

Proof. To show existence of solutions of (S1), we consider first the differential inclusion

$$\frac{dx_i}{dt} \in \mathscr{G}(\mathbf{x}) := \begin{cases} \{x_i g_i(\mathbf{x})\} & \text{if } x_i < 1 \text{ or } g_i(\mathbf{x}) \leq 0 \\ [0, x_i g_i(\mathbf{x})] & \text{if } x_i = 1 \text{ and } g_i(\mathbf{x}) > 0 \\ \{0\} & \text{if } x_i > 1 \text{ and } g_i(\mathbf{x}) > 0. \end{cases} \tag{S2}$$

A differential inclusion differs from a differential equation in that the right hand side returns sets instead of single values or vectors. A Caratheodory solution $\mathbf{x}$ to (S2) is an absolutely continuous map for which (S2) is satisfied almost everywhere. Proposition S2 in [1] states that if

$$\mathscr{F} : [0,\infty) \times \mathbb{R}^n \to \mathfrak{B}(\mathbb{R}^n)$$

is locally bounded and takes non-empty, compact, and convex values, the initial value problem

$$\dot{\mathbf{x}} = \mathscr{F}(t, \mathbf{x}(t)),$$

with $x(0) = \mathbf{x}_0$ has a Caratheodory solution if

1. for each $\mathbf{x} \in \mathbb{R}^n$, the set-valued map $t \to \mathscr{F}(t, \mathbf{x})$ is measurable and
2. for each $t \in \mathbb{R}$, the set-valued map $\mathbf{x} \to \mathscr{F}(t, \mathbf{x})$ is upper semicontinuous.

Here, $\mathfrak{B}(\mathbb{R}^n)$ denotes the set of all subsets of $\mathbb{R}^n$.

Note that $x_i \leq 1$ for all $i \in I$ if $x_i(0) \leq 0$. Therefore,

$$\min\left(0, r_i + \sum_{j \in I, a_{ji} < 0} a_{ji}\right) \;\leq\; x_i g_i(\mathbf{x}) \;\leq\; \max\left(0, r_i + \sum_{j \in I, a_{ji} > 0} a_{ji}\right).$$

Therefore, $\mathscr{G}$ is bounded. Furthermore, $\mathscr{G}$ takes non-empty, compact, and convex values only. Since $\mathscr{G}$ is independent of t, it is measurable in t.

A set-valued map $\mathscr{F}$ is upper semicontinuous if for any argument $\mathbf{x}$ and any series $\mathbf{x}^{(k)} \to \mathbf{x}$, any series $y^{(k)}$ with $y^{(k)} \in \mathscr{F}(\mathbf{x}^{(k)})$ converges to a value $y \in \mathscr{F}(\mathbf{x})$. This is true for $\mathscr{G}$. Thus, the differential inclusion (S2) has a Caratheodory solution.

It remains to be shown that the solutions of (S2) also solve (S1). A classical differential equation is equivalent to a differential inclusion in which the right hand side consist of single-valued sets only. That is, system (S1) is equivalent to

$$\frac{dx_i}{dt} \;\in\; \begin{cases} \{x_i g_i(\mathbf{x})\} & \text{if } x_i < 1 \text{ or } g_i(\mathbf{x}) < 0 \\ \{0\} & \text{else.} \end{cases} \tag{S3}$$

Hence, the only difference of a solution $\mathbf{x}$ of (S2) compared to a solution of (S1) is that for any $i \in I$, $\dot{x}_i$ can take non-zero values for a time of measure larger than zero if $x_i = 1$ and $g_i(\mathbf{x}) > 0$. That is, there may exist a set T of times with a positive Lebesque measure $\lambda(T) > 0$ such that $x_i(t) = 1$, $g_i(\mathbf{x}(t)) > 0$, and $\frac{dx_i}{dt}(t) > 0$ for all $t \in T$.

We argue that this cannot happen in practice. Suppose for contradiction a set T as described above exists. By construction of the Lebesque measure, there is a Borel set $T_B \supseteq T$ with Lebesque measure $\lambda(T_B) = \lambda(T)$. Since T_B is a Borel set, it can be be written as the union of non-empty closed intervals $T_B^{(k)}$, $k \in \mathbb{N}$. Let us consider the interval $T_B^{(1)} = [t_1, t_2]$ and let

$$\varepsilon \;:=\; \min_{t \in T_B^{(1)} \cap T} \frac{dx_i}{dt}(t).$$

We know that $\varepsilon > 0$. We can write

$$x_i(t) \;=\; x_i(t_1) + \int_{t_1}^{t} \frac{dx_i}{dt}(\tau) d\lambda(\tau) \;\geq\; x_i(t_1) + \int_{t_1}^{t} \varepsilon d\lambda(\tau) \;=\; x_i(t_1) + \varepsilon(t - t_1) \;>\; x_i(t_1) \tag{S4}$$

for all $t \in T_B^{(1)}$. We know that $x_i(t_1) = 1$ and $x_i(t) = 1$. This is in contradiction with inequality (S4). Hence, $\frac{dx_i}{dt} = 0$ if $x_i = 1$ and $g_i(\mathbf{x}) > 0$ almost every time and every solution $\mathbf{x}$ of (S2) is also a solution of (S1). □

A.2 Uniqueness of solutions

Reference [1] also contains results on uniqueness of Caratheodory solutions. However, our system does not satisfy their requirements. Hence, we will use a proof based on well-known results for continuous differential equations to show that system (S1) has a unique solution.

Lemma 2. *System (S1) has a unique Caratheodory solution for any initial condition* $\mathbf{x}(0) = \mathbf{x}_0 \in [0,1]^n$.

Proof. We will conduct a proof by contradiction. Suppose there were two distinct solutions $\mathbf{x} \neq \mathbf{y}$ to system (S1) with $\mathbf{x}(0) = \mathbf{y}(0) = \mathbf{x}_0$. As both $\mathbf{x}$ and $\mathbf{y}$ are continuous, there must be an open interval $T = (t_1, t_2)$ with $\mathbf{x}(t_1) = \mathbf{y}(t_1)$ and $\mathbf{x}(t) \neq \mathbf{y}(t)$ for all $t \in T$. Now consider the following system:

$$\frac{d\tilde{x}_i}{dt} \;=\; \begin{cases} \tilde{x}_i g_i(\tilde{\mathbf{x}}) & \text{if } x_i(t_1) < 1 \\ \min(0, \tilde{x}_i g_i(\tilde{\mathbf{x}})) & \text{if } x_i(t_1) = 1 \end{cases} \tag{S5}$$

with $\tilde{\mathbf{x}}(t_1) = \mathbf{x}(t_1)$. Note that the right hand side of system (S5) is continuous, because the condition does not depend on $\tilde{\mathbf{x}}$ or t, and $\min(\cdot,\cdot)$ is a continuous function. In fact, the right hand side of system (S5) is Lipschitz continuous. Therefore, system (S5) has a unique solution.

Let us now consider a solution $\tilde{\mathbf{x}}$ of system (S5). For all $i \in I$, let

$$g_i^{\max} := r_i + \sum_{j\in I, a_{ji}>0} a_{ji},$$
$$T_i^{\min} := \left\{ t \in T \middle| g_i(\tilde{\mathbf{x}}(t)) = 0 \wedge (\exists \tau \in T : \tau < t \wedge g_i(\tilde{\mathbf{x}}_i(\tau)) < 0) \right\},$$

and

$$\tilde{t}_i := \begin{cases} -\frac{\ln x_i(t_1)}{g_i^{\max}} & \text{if } x_i(t_1) < 1 \text{ and } g_i^{\max} > 0 \\ \min T_i^{\min} & \text{if } x_i(t_1) = 1 \text{ and } T_i^{\min} \neq \emptyset \\ \infty & \text{else.} \end{cases}$$

Observe that $\min T_i^{\min}$ exists if $T_i^{\min} \neq \emptyset$, since $g_i(\tilde{\mathbf{x}}(t))$ is continuous. Let

$$\tilde{t} := \min_{i\in I} \tilde{t}_i.$$

Note that $\tilde{t} > t_1$. In the interval $\tilde{T} := [t_1, \tilde{t})$, system (S1) is equivalent to system (S5). This however, contradicts the existence of two distinct solutions to system (S1) in the interval T, because $T \cap \tilde{T} \neq \emptyset$ and system (S5) has a unique solution. Therefore, system (S1) must have a unique solution, too. □

B Average per-capita growth rate and persistence

We show how the non-truncated per-capita growth rate

$$g_i(\mathbf{x}) := r_i + \sum_{j\in I_i} a_{ji} x_j$$

with $i \in I := \{0,\dots,n\}$ and $I_i := I \setminus \{i\}$ is related to survival and extinction of a species i in system (2) (main text). Throughout this appendix, we suppose that the existence assumption from the main text is satisfied; i.e., the limit

$$\bar{\mathbf{x}} = \lim_{T\to\infty} \frac{1}{T} \int_0^T \mathbf{x}(t)dt$$

exists. In our proofs we will consider the actual per-capita growth rate

$$h_i(\mathbf{x}) := \begin{cases} r_i + \sum\limits_{j\in I} a_{ji} x_j & \text{if } x_i < 1 \text{ or } r_i + \sum\limits_j a_{ji} x_j < 0 \\ 0 & \text{else,} \end{cases}$$

and the averages

$$\bar{g}_i := \lim_{T\to\infty} \frac{1}{T} \int_0^T g_i(t)dt = g_i(\bar{\mathbf{x}}) \tag{S6}$$
$$\bar{h}_i := \lim_{T\to\infty} \frac{1}{T} \int_0^T h_i(t)dt. \tag{S7}$$

In equations (S6) and (S7), we write $g_i(t) := g_i(\mathbf{x}(t))$ and $h_i(t) := h_i(\mathbf{x}(t))$ as functions of t. This is only possible if the solution $\mathbf{x}$ to system (2) is already known. That is, the techniques used in this appendix are *not* suitable to *find* a solution to the system.

We rather show that the solution must have certain properties. For example, the solution must satisfy

$$\dot{x}_i(t) = x_i h_i(t)$$

for all $i \in I$. It follows that we can write the solution as

$$x_i(t) = c\exp\left(\int_0^t h_i(\tau)d\tau\right) \tag{S8}$$

with $c = x_i(0)$. We will use this form of the solution to relate it to properties of $\bar{h}_i$ and $\bar{g}_i$.

We proceed by proving two helpful lemmas about $\bar{g}_i$, $\bar{h}_i$, and $\bar{x}_i$. Afterwards, we will show how the sign of $\bar{g}_i$ indicates persistence of a species.

Lemma 3. *It is* $\bar{h}_i \leq 0$.

Proof. Suppose for contradiction that $\bar{h}_i > 0$. By definition of the limit, there is for each $\varepsilon > 0$ a T such that for all $t \geq T$ it is

$$\left|\frac{1}{t}\int_0^t h_i(\tau)d\tau - \bar{h}_i\right| \leq \varepsilon,$$

or equivalently

$$\frac{1}{t}\int_0^t h_i(\tau)d\tau \in \left[\bar{h}_i - \varepsilon, \bar{h}_i + \varepsilon\right].$$

Let us choose $\tilde{\varepsilon} = \frac{\bar{h}_i}{2}$. Then with $t \geq T$,

$$\frac{\bar{h}_i}{2} \leq \frac{1}{t}\int_0^t h_i(\tau)d\tau$$
$$\Longleftrightarrow$$
$$c\exp\left(\frac{\bar{h}_i}{2}t\right) \leq c\exp\left(\int_0^t h_i(\tau)d\tau\right) = x_i(t).$$

That is, $x_i(t)$ diverges. However, $x_i(t)$ cannot increase if $x_i(t) > 1$. Therefore $x_i(t) \leq \max\{1, c\}$. This contradicts divergence. Thus, $\bar{h}_i \leq 0$. □

Lemma 4. *If* $\bar{x}_i = 0$*, then* $\bar{g}_i = \bar{h}_i$.

Proof. Suppose $\bar{x}_i = 0$. Let

$$d_i(t) := g_i(t) - h_i(t)$$

be the difference between the non-truncated and the truncated per-capita growth rate and

$$\bar{d_i} := \lim_{t\to\infty}\frac{1}{t}\int_0^t d_i(\tau)d\tau = \bar{g}_i - \bar{h}_i$$

be its average. Note that

$$d_i(t) = \begin{cases} g_i(t) & \text{if } x(t) \geq 1 \\ -a_{ii}x_i(t) & \text{else,} \end{cases}$$

and that

$$g_i(t) \quad \leq \quad A \; := \; r_i + \sum_{j:\, a_{ji}>0} a_{ji},$$

if $x_j(0) \leq 1$ for all $j \in I$. Recall that $a_{ii} < 0$. Let

$$t_1(T) \quad := \quad \int_0^T \mathrm{I}_{\{1\}}(x(t))dt$$

be the total time between 0 and T where $x(t) = 1$ holds. Here $\mathrm{I}_{\cdot}(\cdot)$ is the indicator function. Define

$$\tilde{x}_i(t) \quad := \quad \mathrm{I}_{\{1\}}(x(t))$$

and note that $\tilde{x}_i(t) \leq x(t)$. From our initial assumptions we get

$$0 \;=\; \bar{x}_i \;=\; \lim_{t\to\infty}\frac{1}{t}\int_0^t x_i(\tau)d\tau \;\geq\; \lim_{t\to\infty}\frac{1}{t}\int_0^t \tilde{x}_i(\tau)d\tau \;=\; \lim_{t\to\infty}\frac{t_1(t)}{t} \;\geq\; 0.$$

Hence,

$$0 \;\leq\; \bar{d}_i \;=\; \lim_{t\to\infty}\frac{1}{t}\int_0^t d_i(\tau)d\tau \;\leq\; \lim_{t\to\infty}\frac{1}{t}\left(t_1(t)A - a_{ii}\int_0^t x_i(t)d\tau\right) \;=\; \lim_{t\to\infty}\frac{t_1(t)}{t}A - a_{ii}\bar{x}_i \;=\; 0,$$

and thus $\bar{g}_i = \bar{h}_i$. □

Theorem. $\bar{x}_i > 0$ *if and only if* $\bar{g}_i > 0$.

Proof. To prove this equivalency, we consider the possible cases $\bar{g}_i > 0$, $\bar{g}_i = 0$, and $\bar{g}_i < 0$ and show that the corresponding $\bar{x}_i$ values satisfy our claim. Since $\bar{g}_i$ must always assume some value, no further proofs are required for the reverse direction.

We start with the proof that $\bar{g}_i > 0$ implies $\bar{x}_i > 0$. Let us assume for contradiction that $\bar{g}_i > 0$ and $\bar{x}_i = 0$. By Lemma 4, it is $\bar{h}_i = \bar{g}_i > 0$. However, by Lemma 3, it is $\bar{h}_i \leq 0$. This is a contradiction, and thus the statement is shown.

We proceed by showing that $\bar{g}_i \leq 0$ implies $\bar{x}_i = 0$. Suppose for contradiction that $\bar{g}_i \leq 0$ and $\bar{x}_i > 0$. Then

$$\begin{aligned}
\bar{h}_i \quad &\leq \quad \lim_{t\to\infty}\frac{1}{t}\int_0^t r_i + \sum_{j\in I} a_{ji}x_j(\tau)d\tau \\
&= \quad \bar{g}_i + a_{ii}\bar{x}_i \;\leq\; a_{ii}\bar{x}_i \;<\; 0
\end{aligned}$$

By the definition of the limit, we can find for each $\varepsilon > 0$ a $T \geq 0$ so that for all $t \geq T$

$$\frac{1}{t}\int_0^t h_i(\tau)d\tau \quad \in \quad \left[\bar{h}_i - \varepsilon, \bar{h}_i + \varepsilon\right].$$

Let us choose $\tilde{\varepsilon} = -\frac{\bar{h}_i}{2}$. Then with $t \geq T$,

$$\frac{\bar{h}_i}{2} \quad \geq \quad \frac{1}{t}\int_0^t h_i(\tau)d\tau$$

$$\Longleftrightarrow$$

$$c\exp\left(\frac{\bar{h}_i}{2}t\right) \quad \geq \quad c\exp\left(\int_0^t h_i(\tau)d\tau\right) \;=\; x_i(t).$$

Since the left hand side converges to 0 exponentially fast, it must be $\bar{x}_i = 0$, which contradicts our initial assumption. Therefore, it must hold $\bar{x}_i = 0$. This concludes the proof. $\square$

C Covariance bounds

In this appendix, we derive bounds for the covariances between the model parameters r_i, a_{ji} and the average species densities $\bar{x}_i$. These covariances play a major role in the approximation of the mean and variance of the average non-truncated per-capita growth rate $\bar{\mathbf{g}}$ with

$$\bar{g}_i(\mathbf{x}) \quad := \quad r_i + \sum_{j \in I_i} a_{ji} \bar{x}_j.$$

In the first subsection, we will introduce notation and basic assumptions used in the later sections. Then we describe the structure of the covariance matrices, before we derive bounds for the covariance terms by exploiting the structure of the covariance matrices and the fact that they must be positive semi-definite.

C.1 Basic assumptions and notation

Throughout this appendix, we suppose that the existence assumption from the main text holds; i.e., the limit $\bar{x}_i := \lim_{T \to \infty} \frac{1}{T} \int_0^T x_i(t) dt$ exists for all $i \in I := \{1, \ldots .n\}$. Recall that in our model $0 \leq \bar{x}_i \leq 1$. Whenever not explicitly defined otherwise, we use the variables i, j, k for arbitrary pair-wise different indices $i, j, k \in I$.

Suppose f is the probability density function of the average species densities $\bar{x}_i$. Let us start by making a note on exchangeability: as the differential equations for the x_i have all the same shape and the random parameters r_i and a_{ji} of these equations are drawn according to the same rules, respectively, the random variables $\bar{x}_i$ are exchangeable. That is, given any permutation $\pi : I \to I$ and an arbitrary vector $\tilde{\mathbf{x}} \in \mathbb{R}^n$, it is $f(\tilde{x}_1, \ldots, \tilde{x}_n) = f\left(\tilde{x}_{\pi(1)}, \ldots, \tilde{x}_{\pi(n)}\right)$. This has consequences on the covariances between the model parameters and the $\bar{x}_i$. For all pair-wise different $i, j, k \in I$, it holds

$$\begin{aligned} \mathrm{Cov}(a_{ji}, \bar{x}_i) &= \alpha, & \mathrm{Cov}(\bar{x}_i, \bar{x}_j) &= \xi, \\ \mathrm{Cov}(a_{ji}, \bar{x}_j) &= \beta, & \mathrm{Cov}(r_i, \bar{x}_i) &= \rho, \\ \mathrm{Cov}(a_{ji}, \bar{x}_k) &= \gamma, & \mathrm{Cov}(r_i, \bar{x}_j) &= \kappa, \end{aligned}$$

whereby the constants on the right hand sides are independent of the indices of the variables on the left hand sides. Note that we ignore the parameters a_{ii} here, as they are irrelevant for the characterization of extinct species and not included in our survival criterion $\bar{g}_i \geq 0$.

Recall that the parameters a_{ji} and r_i are drawn independently from each other. Therefore, the covariances between these parameters are 0. Following the notation in the main text, we write

$$\begin{aligned} \mathbb{E}(a_{ji}) &= \tilde{\mu}_a, & \mathbb{V}(a_{ji}) &= \tilde{\sigma}_a^2, \\ \mathbb{E}(\bar{x}_i) &= \tilde{\mu}_x, & \mathbb{V}(\bar{x}_i) &= \tilde{\sigma}_x^2, \\ \mathbb{E}(r_i) &= \mu_r, & \mathbb{V}(r_i) &= \sigma_r^2. \end{aligned}$$

Note the subtle difference between the parameters $\tilde{\mu}_a$, $\tilde{\mu}_x$, $\tilde{\sigma}_a^2$, and $\tilde{\sigma}_x^2$ defined here and the parameters μ_a, μ_x, σ_a^2, and σ_x^2 defined in the main text. While $\mu_a = \mathbb{E}(a_{ji} | a_{ji} \neq 0)$ and $\sigma_a^2 = \mathbb{V}(a_{ji} | a_{ji} \neq 0)$ refer to the distribution of the interaction coefficients given that the interactions exist (i.e. $a_{ji} \neq 0$), $\tilde{\mu}_a$ and $\tilde{\sigma}_a^2$ refer to the distribution of the interaction coefficients directly. Similarly, $\tilde{\mu}_x$ and $\tilde{\sigma}_x^2$ defined here differ to the μ_x and σ_x^2 specified in the main text in that the latter parameters are conditioned on survival: $\mu_x = \mathbb{E}(\bar{x}_i | \bar{x}_i > 0)$ and $\sigma_x^2 = \mathbb{V}(\bar{x}_i | \bar{x}_i > 0)$.

C.2 Covariance matrices

After these considerations, we can build the covariance matrices. To simplify future computations, it is advisable to consider the covariances between the growth constants r_i and the long time average species densities $\bar{x}_i$ separately from the covariances between the interaction coefficients a_{ji} and the $\bar{x}_i$.

C.2.1 Growth rates and long time average species densities

The covariance matrix C_r for the growth rates r_i and the average species densities $\bar{x}_i$ can be written in block form:

$$C_r = \begin{pmatrix} C_{rr} & C_{rx} \\ C_{rx}^T & C_{xx} \end{pmatrix},$$

whereby the matrix C_{rr} describes the covariances between the parameters r_i, respectively; the matrix C_{rx} contains the covariances between the parameters r_i and the variables $\bar{x}_i$, and the matrix C_{xx} represents the covariances between the average densities $\bar{x}_i$. To describe the blocks, let $\underline{I}$ be the $n \times n$ identity matrix and $\underline{J}$ an $n \times n$ matrix consisting of ones only.

Since all the r_i are drawn independently, the matrix C_{rr} is given by a diagonal matrix:

$$C_{rr} = \sigma_r^2 \underline{I}.$$

The matrix C_{xx} contains the variance $\tilde{\sigma}_x^2$ on the diagonal and is filled with ξ everywhere else:

$$C_{xx} = \left(\tilde{\sigma}_x^2 - \xi\right) \underline{I} + \xi \underline{J}.$$

The matrix C_{rx} contains the covariances $\mathrm{Cov}(r_i, \bar{x}_i) = \rho$ on the diagonal and the covariances $\mathrm{Cov}(r_i, \bar{x}_j) = \kappa$ in the remaining entries:

$$C_{rx} = (\rho - \kappa) \underline{I} + \kappa \underline{J}.$$

C.2.2 Interaction coefficients and long time average species densities

As we did for the covariance between the rates r_i and the densities $\bar{x}_i$, we can write the covariance matrix C_a for the interaction parameters a_{ji} and the densities $\bar{x}_i$ in block matrix form

$$C_a = \begin{pmatrix} C_{aa} & C_{ax} \\ C_{ax}^T & C_{xx} \end{pmatrix},$$

whereby the matrix C_{aa} describes the covariances between the parameters a_{ji}, the matrix C_{ax} contains the covariances between the parameters a_{ji} and the variables $\bar{x}_i$, and the matrix C_{xx} represents the covariances between the average densities $\bar{x}_i$. As above, let $\underline{I}$ be the $n \times n$ identity matrix and $\underline{J}$ an $n \times n$ matrix consisting of ones only. Furthermore, let $\underline{P}$ be the $n \times n$ cyclic permutation matrix. This matrix contains ones at the entries directly below the diagonal and in the upper right corner. That is, for $n = 5$ dimensions, $\underline{P}$ looks as follows:

$$\underline{P} = \begin{pmatrix} 0 & 0 & 0 & 0 & 1 \\ 1 & 0 & 0 & 0 & 0 \\ 0 & 1 & 0 & 0 & 0 \\ 0 & 0 & 1 & 0 & 0 \\ 0 & 0 & 0 & 1 & 0 \end{pmatrix}.$$

Multiplying $\underline{P}$ to a matrix $\underline{M}$ rotates the rows of $\underline{M}$ downwards. That is, the ith row becomes the $(i + 1 \bmod n)$th row. Consequently,

$$\left(\underline{P}^k\right)^T = \underline{P}^{-k} = \underline{P}^{n-k}.$$

Now we can proceed deriving the blocks of C_a. Since the parameters a_{ji} are drawn independently, C_{aa} is a diagonal $n(n-1)\times n(n-1)$ matrix with $\tilde{\sigma}_a^2$ at all diagonal entries. The matrix, C_{xx} is filled everywhere with ξ except for the diagonal, where the matrix is filled with $\tilde{\sigma}_x^2$. That is,

$$C_{xx} = \left(\tilde{\sigma}_x^2-\xi\right)\underline{I}+\xi\underline{J}.$$

The matrix C_{ax} has a more complicated structure. To ease notation, we write the index 0 for the index n if convenient. That is, for example, $a_{i0} := a_{in}$ and $\bar{x}_0 := \bar{x}_n$. Let $\mathbf{A}_i := \left(a_{1,i+1 \bmod n}, a_{2,i+2 \bmod n}, \dots, a_{n,i+n \bmod n}\right)$. For example, if $n=5$, then $\mathbf{A}_2 := (a_{1,3}, a_{2,4}, a_{3,5}, a_{4,1}, a_{5,2})$. Now we can form a vector $\mathbf{A} := (\mathbf{A}_1,\dots,\mathbf{A}_{n-1})$ containing all parameters a_{ji} with $i\neq j$. Note that the entries $\mathbf{A}_1,\dots,\mathbf{A}_{n-1}$ of $\mathbf{A}$ are vectors themselves, which are concatenated to form a long vector. The matrix $C_{ax}^{(i)}$ describing the correlation between the variables in $\mathbf{A}_i$ with the entries of the vector $\bar{\mathbf{x}} = (\bar{x}_1,\dots,\bar{x}_n)$ has the form

$$\operatorname{Cov}(A_i,\bar{\mathbf{x}}) = (\beta-\gamma)\underline{I}+(\alpha-\gamma)\underline{P}^i+\gamma\underline{J}.$$

That is, the diagonal entries of the matrix are given by $\operatorname{Cov}(A_{ij},\bar{x}_j) = \operatorname{Cov}\left(a_{j,i+j \bmod n},\bar{x}_j\right) = \beta$; the entries of the shifted diagonal are $\operatorname{Cov}\left(A_{ij},\bar{x}_{i+j \bmod n}\right) = \operatorname{Cov}\left(a_{j,i+j \bmod n},\bar{x}_{i+j \bmod n}\right) = \alpha$, and the remaining entries of the matrix are γ. With the parameters a_{ji} sorted as in the vector A, the block C_{ax} is given by

$$C_{ax} = \left(\operatorname{Cov}(\mathbf{A}_1,\bar{\mathbf{x}}),\dots,\operatorname{Cov}(\mathbf{A}_{n-1},\bar{\mathbf{x}})\right).$$

C.3 Covariance bounds

Covariance matrices must be positive definite. We can use this property to bound the covariances between the per-capita growth rates, the interaction coefficients, and the long time average species densities.

C.3.1 Growth rates and long time average species densities

Lemma 5. *The characteristic polynomial of the matrix C_r is given by*

$$\begin{aligned}\operatorname{char}C_r = &\left(\lambda^2-\lambda\left(\sigma_r^2+\tilde{\sigma}_x^2+(n-1)\xi\right)+\sigma_r^2\left(\tilde{\sigma}_x^2+(n-1)\xi\right)-(\rho+(n-1)\kappa)^2\right)\\ &\cdot\left(\lambda^2-\lambda\left(\sigma_r^2+\tilde{\sigma}_x^2-\xi\right)+\sigma_r^2\left(\tilde{\sigma}_x^2-\xi\right)-(\rho-\kappa)^2\right)^{n-1}.\end{aligned}$$

Proof. Let $\tilde{C}_r := C_r-\lambda\underline{I}_{2n\times 2n}$, $\tilde{C}_{rr} := C_{rr}-\lambda\underline{I}$, and $\tilde{C}_{xx} := C_{xx}-\lambda\underline{I}$. Using the determinant computation formula for block matrices, we get

$$\det\left(\tilde{C}_r\right) = \det\left(\tilde{C}_{rr}\right)\det\left(\tilde{C}_{xx}-C_{rx}\tilde{C}_{rr}^{-1}C_{rx}^T\right). \tag{S9}$$

Since $\tilde{C}_{rr}$ is a diagonal matrix, it is easy to see that

$$\det\left(\tilde{C}_{rr}\right) = \left(\sigma_r^2-\lambda\right)^n.$$

Furthermore, $\tilde{C}_{rr}^{-1} = \frac{1}{\sigma_r^2-\lambda}\underline{I}$ and therefore

$$\begin{aligned}C_{rx}\tilde{C}_{rr}^{-1}C_{rx}^T &= \frac{1}{\sigma_r^2-\lambda}C_{rx}C_{rx}^T.\\ &= \frac{1}{\sigma_r^2-\lambda}\left((\rho-\kappa)\underline{I}+\kappa\underline{J}\right)\left((\rho-\kappa)\underline{I}+\kappa\underline{J}\right)\\ &= \frac{1}{\sigma_r^2-\lambda}\left((\rho-\kappa)^2\underline{I}+2\kappa(\rho-\kappa)\underline{J}+n\kappa^2\underline{J}\right).\end{aligned}$$

Hence,

$$\tilde{C}_{xx}-C_{rx}\tilde{C}_{rr}^{-1}C_{rx}^T = \left(\tilde{\sigma}_x^2-\xi-\lambda-\frac{(\rho-\kappa)^2}{\sigma_r^2-\lambda}\right)\underline{I}+\left(\xi-\frac{n\kappa^2+2\kappa(\rho-\kappa)}{\sigma_r^2-\lambda}\right)\underline{J}.$$

It is well known (and easy to show by induction) that the characteristic polynomial of a matrix of ones $\underline{J}$ is given by

$$\text{char}(\underline{J}) = \det(\lambda\underline{I} - \underline{J}) = \left(\tilde{\lambda} - n\right)\tilde{\lambda}^{n-1}.$$

Inserting $\tilde{\lambda} = -\frac{a}{b}$ yields

$$\begin{aligned}
\det(a\underline{I} + b\underline{J}) &= (-b)^n \det\left(-\frac{a}{b}\underline{I} - \underline{J}\right) \\
&= (-b)^n \left(-\frac{a}{b} - n\right)\left(-\frac{a}{b}\right)^{n-1} \\
&= (a + nb)\, a^{n-1}.
\end{aligned}$$

Thus,

$$\begin{aligned}
\det\left(\tilde{C}_{xx} - C_{rx}\tilde{C}_{rr}^{-1}C_{rx}^T\right) &= \det\left(\left(\tilde{\sigma}_x^2 - \xi - \lambda - \frac{(\rho-\kappa)^2}{\sigma_r^2 - \lambda}\right)\underline{I} + \left(\xi - \frac{n\kappa^2 + 2\kappa(\rho-\kappa)}{\sigma_r^2 - \lambda}\right)\underline{J}\right) \\
&= \left(\tilde{\sigma}_x^2 - \xi - \lambda - \frac{(\rho-\kappa)^2}{\sigma_r^2 - \lambda} + n\left(\xi - \frac{n\kappa^2 + 2\kappa(\rho-\kappa)}{\sigma_r^2 - \lambda}\right)\right)\left(\tilde{\sigma}_x^2 - \xi - \lambda - \frac{(\rho-\kappa)^2}{\sigma_r^2 - \lambda}\right)^{n-1}
\end{aligned}$$

Inserting the partial results in our formula (S9) yields

$$\begin{aligned}
\text{char}(C_r) &= (-1)^{2n}\det\left(\tilde{C}_r\right) \\
&= \left(\sigma_r^2 - \lambda\right)^n \left(\tilde{\sigma}_x^2 - \xi - \lambda - \frac{(\rho-\kappa)^2}{\sigma_r^2 - \lambda} + n\left(\xi - \frac{n\kappa^2 + 2\kappa(\rho-\kappa)}{\sigma_r^2 - \lambda}\right)\right)\left(\tilde{\sigma}_x^2 - \xi - \lambda - \frac{(\rho-\kappa)^2}{\sigma_r^2 - \lambda}\right)^{n-1} \\
&= \left(\left(\tilde{\sigma}_x^2 - \xi - \lambda\right)\left(\lambda - \sigma_r^2\right) + (\rho-\kappa)^2 + n\left(\xi\left(\lambda - \sigma_r^2\right) + n\kappa^2 + 2\kappa(\rho-\kappa)\right)\right) \\
&\quad \cdot \left(\left(\tilde{\sigma}_x^2 - \xi - \lambda\right)\left(\lambda - \sigma_r^2\right) + (\rho-\kappa)^2\right)^{n-1} \\
&= \left(\lambda^2 - \lambda\left(\sigma_r^2 + \tilde{\sigma}_x^2 + (n-1)\xi\right) + \sigma_r^2\left(\tilde{\sigma}_x^2 + (n-1)\xi\right) - (\rho + (n-1)\kappa)^2\right) \\
&\quad \cdot \left(\lambda^2 - \lambda\left(\sigma_r^2 + \tilde{\sigma}_x^2 - \xi\right) + \sigma_r^2\left(\tilde{\sigma}_x^2 - \xi\right) - (\rho-\kappa)^2\right)^{n-1}
\end{aligned}$$

as desired. □

Corollary 1. *The covariance κ is bounded by terms of order $\mathcal{O}\left(\frac{1}{\sqrt{n}}\right)$.*

Proof. As the matrix C_r is positive semi-definite, all its eigenvalues must be non-negative. These eigenvalues are the zeros of the characteristic polynomial found in Lemma 5. Hence, the eigenvalues satisfy either

$$0 = \lambda^2 - \lambda\left(\sigma_r^2 + \tilde{\sigma}_x^2 + (n-1)\xi\right) + \sigma_r^2\left(\tilde{\sigma}_x^2 + (n-1)\xi\right) - (\rho + (n-1)\kappa)^2 \tag{S10}$$

or

$$0 = \lambda^2 - \lambda\left(\sigma_r^2 + \tilde{\sigma}_x^2 - \xi\right) + \sigma_r^2\left(\tilde{\sigma}_x^2 - \xi\right) - (\rho-\kappa)^2. \tag{S11}$$

The smaller solution of equation (S11) is given by

$$\begin{aligned}
\lambda_{min,2} &= \frac{1}{2}\left(\sigma_r^2+\tilde{\sigma}_x^2-\xi\right)-\sqrt{\frac{1}{4}\left(\sigma_r^2+\tilde{\sigma}_x^2-\xi\right)^2-\sigma_r^2\left(\tilde{\sigma}_x^2-\xi\right)+\left(\rho-\kappa\right)^2} \\
&\geq 0 \\
\Longleftrightarrow & \\
0 &\geq -\sigma_r^2\left(\tilde{\sigma}_x^2-\xi\right)+\left(\rho-\kappa\right)^2 \\
\Longleftrightarrow & \\
\left(\rho-\kappa\right)^2 &\leq \sigma_r^2\left(\tilde{\sigma}_x^2-\xi\right).
\end{aligned}$$

Hence, $\rho \in \left[\kappa \pm \sigma_r\sqrt{\tilde{\sigma}_x^2-\xi}\right]$. Note that $\tilde{\sigma}_x^2 \geq \xi$ by the Cauchy-Schwarz inequality. Let us now consider the smaller solution of equation (S10):

$$\begin{aligned}
\lambda_{min,1} &= \frac{1}{2}\left(\sigma_r^2+\tilde{\sigma}_x^2+(n-1)\,\xi\right)-\sqrt{\frac{1}{4}\left(\sigma_r^2+\tilde{\sigma}_x^2+(n-1)\,\xi\right)^2-\sigma_r^2\left(\tilde{\sigma}_x^2+(n-1)\,\xi\right)+\left(\rho+(n-1)\,\kappa\right)^2} \\
&\geq 0 \\
\Longleftrightarrow & \\
0 &\geq -\sigma_r^2\left(\tilde{\sigma}_x^2+(n-1)\,\xi\right)+\left(\rho+(n-1)\,\kappa\right)^2 \\
\Longleftrightarrow & \\
\left(\rho+(n-1)\,\kappa\right)^2 &\leq \sigma_r^2\left(\tilde{\sigma}_x^2+(n-1)\,\xi\right) \\
&\leq n\sigma_r^2\tilde{\sigma}_x^2
\end{aligned}$$

Hence,

$$(n-1)\,\kappa \in \left[-\rho-\sqrt{n}\sigma_r\sigma_{\bar{x}},-\rho+\sqrt{n}\sigma_r\sigma_{\bar{x}}\right].$$

Inserting our bounds for ρ yields

$$\begin{aligned}
(n-1)\,\kappa &\in \left[-\kappa-\sigma_r\sqrt{\tilde{\sigma}_x^2-\xi}-\sqrt{n}\sigma_r\sigma_{\bar{x}},-\kappa+\sigma_r\sqrt{\tilde{\sigma}_x^2-\xi}+\sqrt{n}\sigma_r\sigma_{\bar{x}}\right] \\
\Longleftrightarrow & \\
n\kappa &\in \left[-\sigma_r\sqrt{\tilde{\sigma}_x^2-\xi}-\sqrt{n}\sigma_r\sigma_{\bar{x}},\sigma_r\sqrt{\tilde{\sigma}_x^2-\xi}+\sqrt{n}\sigma_r\sigma_{\bar{x}}\right].
\end{aligned}$$

With $\tilde{\sigma}_x^2 \geq \xi$, we obtain $|\kappa| \leq \sigma_r\sigma_{\bar{x}}\left(\frac{1}{n}+\frac{1}{\sqrt{n}}\right)$.

As $\bar{x} \in [0,1]$, we know by Popoviciu's inequality on variances that $\sigma_{\bar{x}} \leq \frac{1}{2}$. Furthermore, σ_r^2 is a known constant. Hence, the absolute value of κ decreases at least in $\mathscr{O}\left(\frac{1}{\sqrt{n}}\right)$ as n increases. □

C.3.2 Interaction coefficients and long time average species densities

Lemma 6. *The characteristic polynomial of the matrix C_a is given by*

$$\begin{aligned}
\mathrm{char}C_a = {}& \left(\lambda-\sigma_r^2\right)^{(n-1)^2-1}\left(\left(\tilde{\sigma}_a^2-\lambda\right)\left(\tilde{\sigma}_x^2-\lambda+(n-1)\,\xi\right)-(n-1)\left(\beta+\alpha+(n-2)\,\gamma\right)^2\right) \\
& \cdot\left(\left(\sigma_r^2-\lambda\right)\left(\tilde{\sigma}_x^2-\lambda-\xi\right)-\left((n-2)\left((\alpha-\gamma)^2+(\beta-\gamma)^2\right)+(\alpha-\beta)^2\right)\right)^{n-1}
\end{aligned}$$

Proof. We proceed similar to the proof for Lemma 5. Let $\tilde{C}_a := C_a-\lambda\underline{I}_{n^2\times n^2}$, $\tilde{C}_{aa} := C_{aa}-\lambda\underline{I}_{n(n-1)\times n(n-1)}$, and $\tilde{C}_{xx} := C_{xx}-\lambda\underline{I}$. Using the determinant formula for block matrices, we get

$$\det\left(\tilde{C}_a\right) = \det\left(\tilde{C}_{aa}\right)\det\left(\tilde{C}_{xx}-C_{ax}\tilde{C}_{xx}^{-1}C_{ax}^T\right). \tag{S12}$$

Since $\tilde{C}_{aa}$ is a diagonal matrix, it is

$$\det\left(\tilde{C}_{aa}\right) = \left(\tilde{\sigma}_a^2 - \lambda\right)^n.$$

Furthermore, $\tilde{C}_{aa}^{-1} = \frac{1}{\tilde{\sigma}_a^2-\lambda}\underline{I}_{n(n-1)\times n(n-1)}$ and therefore

$$\begin{aligned}
C_{ax}\tilde{C}_{aa}^{-1}C_{ax}^T &= \frac{1}{\tilde{\sigma}_a^2-\lambda}C_{ax}C_{ax}^T.\\
&= \frac{1}{\tilde{\sigma}_a^2-\lambda}\left(\mathrm{Cov}(A_1,\bar{\mathbf{x}}),\ldots,\mathrm{Cov}(A_{n-1},\bar{\mathbf{x}})\right)\cdot\left(\mathrm{Cov}(A_1,\bar{\mathbf{x}}),\ldots,\mathrm{Cov}(A_{n-1},\bar{\mathbf{x}})\right)^T\\
&= \frac{1}{\tilde{\sigma}_a^2-\lambda}\sum_{i=1}^{n-1}\mathrm{Cov}(A_i,\bar{\mathbf{x}})\cdot\mathrm{Cov}(A_i,\bar{\mathbf{x}})^T.
\end{aligned}$$

It is

$$\begin{aligned}
\mathrm{Cov}(A_i,\bar{\mathbf{x}})\cdot\mathrm{Cov}(A_i,\bar{\mathbf{x}})^T &= \left((\beta-\gamma)\underline{I}+(\alpha-\gamma)\underline{P}^i+\gamma\underline{J}\right)\cdot\left((\beta-\gamma)\underline{I}+(\alpha-\gamma)\underline{P}^i+\gamma\underline{J}\right)^T\\
&= \left((\beta-\gamma)\underline{I}+(\alpha-\gamma)\underline{P}^i+\gamma\underline{J}\right)\cdot\left((\beta-\gamma)\underline{I}+(\alpha-\gamma)\underline{P}^{n-i}+\gamma\underline{J}\right)\\
&= (\beta-\gamma)^2\underline{I}+(\alpha-\gamma)^2\underbrace{\underline{P}^i\underline{P}^{n-i}}_{\underline{I}}+\gamma^2\underbrace{\underline{J}\underline{J}}_{n\underline{J}}\\
&\quad+(\beta-\gamma)(\alpha-\gamma)\left(\underline{P}^i+\underline{P}^{n-i}\right)+2(\beta-\gamma)\gamma\underline{J}\\
&\quad+(\alpha-\gamma)\gamma\Big(\underbrace{\underline{P}^i\underline{J}}_{\underline{J}}+\underbrace{\underline{P}^i\underline{J}}_{\underline{J}}\Big)\\
&= \left((\alpha-\gamma)^2+(\beta-\gamma)^2\right)\underline{I}+\left((n-4)\gamma+2(\alpha+\beta)\right)\gamma\underline{J}\\
&\quad+(\beta-\gamma)(\alpha-\gamma)\left(\underline{P}^i+\underline{P}^{n-i}\right).
\end{aligned}$$

Hence,

$$\begin{aligned}
C_{ax}\tilde{C}_{aa}^{-1}C_{ax}^T = \frac{1}{\tilde{\sigma}_a^2-\lambda}\Bigg(&(n-1)\left((\alpha-\gamma)^2+(\beta-\gamma)^2\right)\underline{I}\\
&+(n-1)\left((n-4)\gamma+2(\alpha+\beta)\right)\gamma\underline{J}\\
&+(\beta-\gamma)(\alpha-\gamma)\sum_{i=1}^{n-1}\left(\underline{P}^i+\underline{P}^{n-i}\right)\Bigg).
\end{aligned}$$

Note that

$$\begin{aligned}
\sum_{i=1}^{n-1}\left(\underline{P}^i+\underline{P}^{n-i}\right) &= 2\sum_{i=1}^{n-1}\underline{P}^i\\
&= 2\left(\underline{J}-\underline{I}\right),
\end{aligned}$$

and thus

$$\begin{aligned}
C_{ax}\tilde{C}_{aa}^{-1}C_{ax}^T = \frac{1}{\tilde{\sigma}_a^2-\lambda}\Bigg(&\left((n-1)\left((\alpha-\gamma)^2+(\beta-\gamma)^2\right)-2(\beta-\gamma)(\alpha-\gamma)\right)\underline{I}\\
&+\left((n-1)\left((n-4)\gamma+2(\alpha+\beta)\right)\gamma+2(\beta-\gamma)(\alpha-\gamma)\right)\underline{J}\Bigg).
\end{aligned}$$

Consequently,

$$\begin{aligned}
\tilde{C}_{xx}-C_{ax}\tilde{C}_{aa}^{-1}C_{ax}^T = &\left(\tilde{\sigma}_x^2-\xi-\lambda-\frac{n-1}{\sigma_r^2-\lambda}\left((\alpha-\gamma)^2+(\beta-\gamma)^2\right)+\frac{2(\beta-\gamma)(\alpha-\gamma)}{\sigma_r^2-\lambda}\right)\underline{I}\\
&+\left(\xi-\frac{(n-1)\gamma}{\sigma_r^2-\lambda}\left((n-4)\gamma+2(\alpha+\beta)\right)-\frac{2(\beta-\gamma)(\alpha-\gamma)}{\sigma_r^2-\lambda}\right)\underline{J}
\end{aligned}$$

Recall from the proof of Lemma 5 that for $a, b \in \mathbb{R}$,

$$\det\left(a\underline{I} + b\underline{J}\right) \;=\; (a+nb)\, a^{n-1}.$$

Inserting and simplifying yields

$$\begin{aligned}\det\left(\tilde{C}_{xx} - C_{ax}\tilde{C}_{aa}^{-1}C_{ax}^{T}\right) \;=\;& \left(\tilde{\sigma}_x^2 - \lambda + (n-1)\,\xi - \frac{n-1}{\tilde{\sigma}_a^2 - \lambda}\left(\alpha + \beta + (n-2)\,\gamma\right)^2\right) \\ &\cdot \left(\tilde{\sigma}_x^2 - \lambda - \xi - \frac{1}{\sigma_r^2 - \lambda}\left((n-2)\left((\beta-\gamma)^2 + (\alpha-\gamma)^2\right) + (\alpha-\beta)^2\right)\right)^{n-1}\end{aligned}$$

and

$$\begin{aligned}\mathrm{char}\left(C_a\right) \;=\;& (-1)^{n^2}\det\left(\tilde{C}_a\right) \\ =\;& (-1)^{n^2}\left(\tilde{\sigma}_a^2 - \lambda\right)^{n(n-1)}\left(\tilde{\sigma}_x^2 - \lambda + (n-1)\,\xi - \frac{n-1}{\tilde{\sigma}_a^2 - \lambda}\left(\alpha + \beta + (n-2)\,\gamma\right)^2\right) \\ &\cdot\left(\tilde{\sigma}_x^2 - \lambda - \xi - \frac{1}{\sigma_r^2 - \lambda}\left((n-2)\left((\beta-\gamma)^2 + (\alpha-\gamma)^2\right) + (\alpha-\beta)^2\right)\right)^{n-1} \\ =\;& \left(\lambda - \tilde{\sigma}_a^2\right)^{(n-1)^2-1}\left(\left(\tilde{\sigma}_a^2 - \lambda\right)\left(\tilde{\sigma}_x^2 - \lambda + (n-1)\,\xi\right) - (n-1)\left(\alpha+\beta+(n-2)\,\gamma\right)^2\right) \\ &\cdot\left(\left(\tilde{\sigma}_a^2 - \lambda\right)\left(\tilde{\sigma}_x^2 - \lambda - \xi\right) - (n-2)\left((\beta-\gamma)^2 + (\alpha-\gamma)^2\right) - (\alpha-\beta)^2\right)^{n-1}\end{aligned}$$

as claimed. □

Corollary 2. *The covariances α, β, and γ satisfy $\alpha = \mathcal{O}\left(\frac{1}{\sqrt{n}}\right)$, $\beta = \mathcal{O}\left(\frac{1}{\sqrt{n}}\right)$, and $\gamma = \mathcal{O}\left(\frac{1}{n}\right)$.*

Proof. The proof is analogous to the proof of Corollary 1. As the matrix C_a is positive semi-definite, all its eigenvalues are non-negative. These eigenvalues are the zeros of the characteristic polynomial found in Lemma 6. Hence, the eigenvalues satisfy either

$$\lambda \;=\; \tilde{\sigma}_a^2,$$

or

$$\begin{aligned}0 \;=\;& \left(\tilde{\sigma}_a^2 - \lambda\right)\left(\tilde{\sigma}_x^2 - \lambda + (n-1)\,\xi\right) - (n-1)\left(\alpha+\beta+(n-2)\,\gamma\right)^2 \\ =\;& \lambda^2 - \lambda\left(\tilde{\sigma}_a^2 + \tilde{\sigma}_x^2 + (n-1)\,\xi\right) + \tilde{\sigma}_a^2\tilde{\sigma}_x^2 + (n-1)\left(\tilde{\sigma}_a^2\xi - \left(\alpha+\beta+(n-2)\,\gamma\right)^2\right),\end{aligned} \tag{S13}$$

or

$$\begin{aligned}0 \;=\;& \left(\tilde{\sigma}_a^2 - \lambda\right)\left(\tilde{\sigma}_x^2 - \lambda - \xi\right) - \left((n-2)\left((\alpha-\gamma)^2 + (\beta-\gamma)^2\right) + (\alpha-\beta)^2\right) \\ =\;& \lambda^2 - \lambda\left(\tilde{\sigma}_a^2 + \tilde{\sigma}_x^2 - \xi\right) + \tilde{\sigma}_a^2\left(\tilde{\sigma}_x^2 + \xi\right) - (n-2)\left((\beta-\gamma)^2 + (\alpha-\gamma)^2\right) - (\alpha-\beta)^2.\end{aligned} \tag{S14}$$

It is clear that the variance $\tilde{\sigma}_a^2$ is non-negative. Equation (S13) has no negative solution if and only if

$$\begin{aligned}0 \;\leq\;& \tilde{\sigma}_a^2\tilde{\sigma}_x^2 + (n-1)\left(\tilde{\sigma}_a^2\xi - \left(\alpha+\beta+(n-2)\,\gamma\right)^2\right) \\ &\Longleftrightarrow \\ \left(\alpha+\beta+(n-2)\,\gamma\right)^2 \;\leq\;& \frac{\tilde{\sigma}_a^2\tilde{\sigma}_x^2}{n-1} + \tilde{\sigma}_a^2\xi \\ &\Longleftrightarrow \\ \gamma \;\in\;& \left[-\frac{\alpha+\beta}{n-2} \pm \frac{1}{n-2}\sqrt{\frac{\tilde{\sigma}_a^2\tilde{\sigma}_x^2}{n-1} + \tilde{\sigma}_a^2\xi}\right]\end{aligned} \tag{S15}$$

Secondly, equation (S14) has non-negative solutions only if and only if

$$
\begin{aligned}
0 &\leq \tilde{\sigma}_a^2\left(\tilde{\sigma}_x^2+\xi\right)-(n-2)\left((\alpha-\gamma)^2+(\beta-\gamma)^2\right)-(\alpha-\beta)^2 \\
&\Longleftrightarrow \\
(\alpha-\gamma)^2+(\beta-\gamma)^2+\frac{(\alpha-\beta)^2}{n-2} &\leq \frac{\tilde{\sigma}_a^2\left(\tilde{\sigma}_x^2+\xi\right)}{n-2}.
\end{aligned}
$$

In particular,

$$
\begin{aligned}
(\alpha-\gamma)^2 &\leq \frac{\tilde{\sigma}_a^2\left(\tilde{\sigma}_x^2+\xi\right)}{n-2} \\
(\beta-\gamma)^2 &\leq \frac{\tilde{\sigma}_a^2\left(\tilde{\sigma}_x^2+\xi\right)}{n-2} \\
\Longrightarrow & \\
\alpha &\in \left[\gamma \pm \sqrt{\frac{\tilde{\sigma}_a^2\left(\tilde{\sigma}_x^2+\xi\right)}{n-2}}\right] \qquad \text{(S16)} \\
\beta &\in \left[\gamma \pm \sqrt{\frac{\tilde{\sigma}_a^2\left(\tilde{\sigma}_x^2+\xi\right)}{n-2}}\right]. \qquad \text{(S17)}
\end{aligned}
$$

From here it is a short exercise to bound the absolute values of α, β, and γ by terms dependent on constants and n. Inserting (S15) in (S16) yields

$$
\begin{aligned}
\alpha &\in \left[\gamma \pm \sqrt{\frac{\tilde{\sigma}_a^2\left(\tilde{\sigma}_x^2+\xi\right)}{n-2}}\right] \\
&\subseteq \left[-\frac{\alpha+\beta}{n-2} \pm \left(\frac{1}{n-2}\sqrt{\frac{\tilde{\sigma}_a^2\tilde{\sigma}_x^2}{n-1}+\tilde{\sigma}_a^2\xi}+\sqrt{\frac{\tilde{\sigma}_a^2\left(\tilde{\sigma}_x^2+\xi\right)}{n-2}}\right)\right] \\
&\Longleftrightarrow \\
\alpha\left(1+\frac{1}{n-2}\right) &\in \left[-\frac{\beta}{n-2} \pm \left(\frac{1}{n-2}\sqrt{\frac{\tilde{\sigma}_a^2\tilde{\sigma}_x^2}{n-1}+\tilde{\sigma}_a^2\xi}+\sqrt{\frac{\tilde{\sigma}_a^2\left(\tilde{\sigma}_x^2+\xi\right)}{n-2}}\right)\right] \\
&\Longrightarrow \\
\alpha &\in \left[-\frac{\beta}{n-2} \pm \left(\frac{1}{n-2}\sqrt{\frac{\tilde{\sigma}_a^2\tilde{\sigma}_x^2}{n-1}+\tilde{\sigma}_a^2\xi}+\sqrt{\frac{\tilde{\sigma}_a^2\left(\tilde{\sigma}_x^2+\xi\right)}{n-2}}\right)\right].
\end{aligned}
$$

Repeating this procedure and inserting the result gives us

$$
\begin{aligned}
\beta &\in \left[\gamma \pm \sqrt{\frac{\tilde{\sigma}_a^2\,(\tilde{\sigma}_x^2+\xi)}{n-2}}\right]. \\
&\subseteq \left[-\frac{\alpha+\beta}{n-2} \pm \left(\frac{1}{n-2}\sqrt{\frac{\tilde{\sigma}_a^2\tilde{\sigma}_x^2}{n-1}+\tilde{\sigma}_a^2\xi}+\sqrt{\frac{\tilde{\sigma}_a^2\,(\tilde{\sigma}_x^2+\xi)}{n-2}}\right)\right] \\
&\subseteq \left[-\frac{(n-3)\,\beta}{(n-2)^2} \pm \frac{n-1}{n-2}\left(\frac{1}{n-2}\sqrt{\frac{\tilde{\sigma}_a^2\tilde{\sigma}_x^2}{n-1}+\tilde{\sigma}_a^2\xi}+\sqrt{\frac{\tilde{\sigma}_a^2\,(\tilde{\sigma}_x^2+\xi)}{n-2}}\right)\right] \\
&\iff \\
\beta\left(1+\frac{n-3}{(n-2)^2}\right) &\in \left[\pm\frac{n-1}{n-2}\left(\frac{1}{n-2}\sqrt{\frac{\tilde{\sigma}_a^2\tilde{\sigma}_x^2}{n-1}+\tilde{\sigma}_a^2\xi}+\sqrt{\frac{\tilde{\sigma}_a^2\,(\tilde{\sigma}_x^2+\xi)}{n-2}}\right)\right] \\
&\implies \\
\beta &\in \left[\pm\frac{n-1}{n-2}\left(\frac{1}{n-2}\sqrt{\frac{\tilde{\sigma}_a^2\tilde{\sigma}_x^2}{n-1}+\tilde{\sigma}_a^2\xi}+\sqrt{\frac{\tilde{\sigma}_a^2\,(\tilde{\sigma}_x^2+\xi)}{n-2}}\right)\right].
\end{aligned}
$$

Since $|\xi| \leq \tilde{\sigma}_x^2$ is bounded, we obtain after backsubstitution $\alpha = \mathscr{O}\left(\frac{1}{\sqrt{n}}\right)$, $\beta = \mathscr{O}\left(\frac{1}{\sqrt{n}}\right)$, and $\gamma = \mathscr{O}\left(\frac{1}{n}\right)$ as desired. □

Corollary 3. *It is* $\mathrm{Cov}\left(a_{ji}^2, \bar{x}_i^2\right) = \mathscr{O}\left(\frac{1}{\sqrt{n}}\right)$, $\mathrm{Cov}\left(a_{ji}^2, \bar{x}_j^2\right) = \mathscr{O}\left(\frac{1}{\sqrt{n}}\right)$, *and* $\mathrm{Cov}\left(a_{ji}^2, \bar{x}_k^2\right) = \mathscr{O}\left(\frac{1}{n}\right)$.

Proof. The covariance matrix for the variables a_{ji}^2 and $\bar{x}_k^2$ with $i,k \in I$, $j \in I_i$ has the same structure as the covariance matrix for the variables a_{ji} and $\bar{x}_k$ as constructed in section C.2.2. Hence, Lemma 6 holds with appropriate relabelling of the constants. The distribution of a_{ji}^2 along with its mean and variance are known. Furthermore, $\bar{x}_k^2 \in [0,1]$, which implies that $\mathbb{V}\left(\bar{x}_k^2\right) \leq \frac{1}{4}$. Thus, the proof of this corollary is analog to the proof of Corollary 2. □

D Approximation of mean and variance of the average non-truncated per-capita growth rate $\bar{g}_i$

In this appendix, we derive approximations for the mean and the variance of the non-truncated per-capita growth rate $\bar{g}_i = r_i + \sum_{j \in I_i} a_{ji}\bar{x}_j$.

D.1 Notation and preliminaries

In accordance with our notation in Appendix C, let us write for pair-wise distinct $i,j,k \in I := \{1,\ldots,n\}$

$$
\begin{aligned}
\mathrm{Cov}(a_{ji}, \bar{x}_i) &= \alpha, & \mathrm{Cov}(\bar{x}_i, \bar{x}_j) &= \xi, \\
\mathrm{Cov}(a_{ji}, \bar{x}_j) &= \beta, & \mathrm{Cov}(r_i, \bar{x}_i) &= \rho, \\
\mathrm{Cov}(a_{ji}, \bar{x}_k) &= \gamma, & \mathrm{Cov}(r_i, \bar{x}_j) &= \kappa,
\end{aligned}
$$

and

$$
\begin{aligned}
\mathbb{E}(a_{ji}) &= \tilde{\mu}_a, & \mathbb{V}(a_{ji}) &= \tilde{\sigma}_a^2, \\
\mathbb{E}(\bar{x}_i) &= \tilde{\mu}_x, & \mathbb{V}(\bar{x}_i) &= \tilde{\sigma}_x^2, \\
\mathbb{E}(r_i) &= \mu_r, & \mathbb{V}(r_i) &= \sigma_r^2.
\end{aligned}
$$

Furthermore, define $\beta_2 := \mathrm{Cov}\left(a_{ji}^2, \bar{x}_j^2\right)$.

Let $\Phi_i = \mathrm{I}_{(0,1]}(\bar{x}_i)$ be the random variable modelling the survival of a species i. Here, $\mathrm{I}_{\cdot}(\cdot)$ denotes the indicator function. Let furthermore $\chi_i = \bar{x}_i \big| (\bar{x}_i > 0)$ be a random variable modelling the average density of species i if it is known to survive. We can write $\bar{x}_i = \Phi_i \chi_i$ and regard the random variables χ_i and Φ_i as independent from each other. Consequently,

$$\mathbb{E}(\bar{x}_i) \;=\; \tilde{\mu}_x \;=\; \mathbb{E}(\Phi_i)\mathbb{E}(\chi_i) = P\mu_x \tag{S18}$$

and

$$\begin{aligned}\mathbb{V}(\bar{x}_i) &= \tilde{\sigma}_x^2 \\ &= \mathbb{E}(\chi_i)^2\mathbb{V}(\Phi_i) + \mathbb{V}(\chi_i)\mathbb{E}(\Phi_i)^2 + \mathbb{V}(\chi_i)\mathbb{V}(\Phi_i) \\ &= \mu_x^2 P(1-P) + \sigma_x^2 P(1-P) + \sigma_x^2 P^2 \end{aligned} \tag{S19}$$

By Popoviciu's inequality on variances, it is $\sigma_x^2 \leq \mu_x(1-\mu_x)$ and we may estimate $\sigma_x^2 \approx \omega_1 \mu_x (1-\mu_x)$ with some constant ω_1. Provided the intra-specific competition does not hinder species from growing to the sharp density bound, this approach leads to good quantitative estimates of σ_x^2 if we choose $\omega_1 = \frac{1}{2}$ (see Appendix E). For the case of strong intra-specific competition. we suggest a different estimate for σ_x^2 in Approximation 2 in the main text. However, without loss of generality, we will proceed with the estimate given above. In the case of strong self-damping, our estimates can be adjusted easily.

We note that σ_x^2 is often small, as surviving species either are either likely to grow to their capacity, implying that μ_x is large, or may be kept at a small density by intra-specific competition. Therefore, it can be of advantage to neglect σ_x^2 completely and regard $\bar{x}_i$ as a scaled Bernoulli random variable if we are not seeking for quantitative estimates of the variance $\tilde{\sigma}_x^2$ but rather for approximations with the right order of magnitude. That is, we may approximate $\bar{x}_i$ as

$$\bar{x}_i \;\approx\; \mu_x \Phi_i \tag{S20}$$

and hence

$$\tilde{\sigma}_x^2 \;\approx\; \mu_x^2 P(1-P). \tag{S21}$$

D.2 Mean

In the main text, we have already noted that

$$\begin{aligned}\mathbb{E}(\bar{g}_i) &= \mathbb{E}\left(r_i + \sum_{j\in I_i} a_{ji}\bar{x}_j\right) \\ &= \mathbb{E}(r_i) + \sum_{j\in I_i} \mathbb{E}(a_{ji}\bar{x}_j) \\ &= \mathbb{E}(r_i) + \sum_{j\in I_i} \left(\mathbb{E}(a_{ji})\mathbb{E}(\bar{x}_j) + \mathrm{Cov}(a_{ji},\bar{x}_j)\right) \\ &= \mu_r + (n-1)c\mu_a P\mu_x + (n-1)\mathrm{Cov}(a_{ji},\bar{x}_j).\end{aligned}$$

In appendix C we have shown that $\mathrm{Cov}(a_{ji},\bar{x}_j) = \mathscr{O}\left(\frac{1}{\sqrt{n}}\right)$. Hence, we may neglect the covariance term:

$$\mathbb{E}(\bar{g}_i) \approx \mu_r + (n-1)c\mu_a P\mu_x.$$

If $c\mu_a P\mu_x \approx 0$, then the covariance terms can become significant. This cannot happen if $\mu_a > 0$, as this results in large values of P and μ_x. Furthermore, if $c = 0$, all variables are independent of each other and the covariance is 0. Nonetheless, the lack of precise covariance terms can distract our model precision if $\mu_a = 0$ or if P is small. Below we will show, however, that the covariance is small in these cases.

D.3 Variance

Now we derive the variance of $\bar{g}_i$. We obtain

$$
\begin{aligned}
\mathbb{V}(\bar{g}_i) &= \mathbb{V}\left(r_i + \sum_{j\in I_i} a_{ji}\bar{x}_j\right) \\
&= \sigma_r^2 + (n-1)\,\mathbb{V}(a_{ji}\bar{x}_j) + 2\,(n-1)\,\mathrm{Cov}(r_i, a_{ji}\bar{x}_j) \\
&\quad + (n-2)\,(n-1)\,\mathrm{Cov}(a_{ji}\bar{x}_j, a_{ki}\bar{x}_k). \qquad \text{(S22)}
\end{aligned}
$$

Thereby,

$$
\begin{aligned}
\mathbb{V}(a_{ji}\bar{x}_j) &= \beta_2 + \left(\tilde{\mu}_a^2 + \tilde{\sigma}_a^2\right)\left(\tilde{\mu}_x^2 + \tilde{\sigma}_x^2\right) - \left(\beta + \tilde{\mu}_a\tilde{\mu}_x\right)^2 \\
&= \tilde{\mu}_a^2\tilde{\sigma}_x^2 + \tilde{\sigma}_a^2\tilde{\mu}_x^2 + \tilde{\sigma}_a^2\tilde{\sigma}_x^2 - 2\tilde{\mu}_a\tilde{\mu}_x\beta - \beta^2 + \beta_2.
\end{aligned}
$$

Inserting (S18) and (S19), using $\sigma_x^2 \approx \omega\mu_x\,(1-\mu_x)$, and applying the covariance bounds found in appendix C, we obtain

$$
\begin{aligned}
\mathbb{V}(a_{ji}\bar{x}_j) &= c^2\mu_a^2\left(\mu_x^2 P\,(1-P) + \sigma_x^2 P\,(1-P) + P^2\sigma_x^2\right) + P^2\mu_x^2\left(\mu_a^2 c\,(1-c) + \sigma_a^2 c\,(1-c) + c^2\sigma_a^2\right) \\
&\quad + \left(\mu_x^2 P\,(1-P) + \sigma_x^2 P\,(1-P) + P^2\sigma_x^2\right)\left(\mu_a^2 c\,(1-c) + \sigma_a^2 c\,(1-c) + c^2\sigma_a^2\right) - 2c\mu_a P\mu_x\beta - \beta^2 + \beta_2 \\
&= cP\left(\mu_x^2 + \sigma_x^2\right)\left(\mu_a^2 + \sigma_a^2\right) - c^2P^2\mu_x^2\mu_a^2 + \mathscr{O}\left(\frac{1}{\sqrt{n}}\right) \\
&\approx cP\mu_x\left(\left(\omega + \mu_x\,(1-\omega)\right)\left(\mu_a^2 + \sigma_a^2\right) - cP\mu_x\mu_a^2\right) + \mathscr{O}\left(\frac{1}{\sqrt{n}}\right) \\
\overset{\text{with } \omega=2}{=}\; & cP\mu_x\left(\frac{1+\mu_x}{2}\left(\mu_a^2 + \sigma_a^2\right) - cP\mu_x\mu_a^2\right) + \mathscr{O}\left(\frac{1}{\sqrt{n}}\right)
\end{aligned}
$$

For the covariance terms, we obtain (see [3])

$$
\mathrm{Cov}(r_i, a_{ji}\bar{x}_j) = \tilde{\mu}_a\kappa + 0\tilde{\mu}_x + \mathbb{E}(\Delta r_i\Delta a_{ji}\Delta x_j) \qquad \text{(S23)}
$$

and

$$
\begin{aligned}
\mathrm{Cov}(a_{ji}x_j, a_{ki}x_k) &= \tilde{\mu}_a^2\xi + 2\tilde{\mu}_a\tilde{\mu}_x\gamma + \mathbb{E}(\Delta a_{ji}\Delta a_{ki}\Delta x_j\Delta x_k) \\
&\quad + 2\tilde{\mu}_a\mathbb{E}(\Delta a_{ji}\Delta x_j\Delta x_k) + 2\tilde{\mu}_x\mathbb{E}(\Delta a_{ji}\Delta a_{ki}\Delta x_j) - \beta^2. \qquad \text{(S24)}
\end{aligned}
$$

Here, the Δ denotes the centralized versions of the respective random variables. That is, for a random variable Y, it is $\Delta Y = Y - \mathbb{E}(Y)$. The higher moments in equations (S23) and (S24) are hard to compute. However, it is a common practice to approximate covariances ignoring these higher moments. Hence, we can write

$$
\mathrm{Cov}(r_i, a_{ji}\bar{x}_j) = \mathscr{O}\left(\frac{1}{\sqrt{n}}\right)
$$

and

$$
\mathrm{Cov}(a_{ji}x_j, a_{ki}x_k) = \mathscr{O}\left(\xi + \frac{1}{n}\right)
$$

applying the covariance bounds we found in appendix C. Consequently,

$$
\begin{aligned}
\mathbb{V}(\bar{g}_i) &= \sigma_r^2 + (n-1)\,cP\mu_x\left(\frac{1+\mu_x}{2}\left(\mu_a^2 + \sigma_a^2\right) - cP\mu_x\mu_a^2\right) \\
&\quad + (n-2)\,(n-1)\,c^2\mu_a^2\xi + \mathscr{O}(n).
\end{aligned}
$$

For a useful variance estimate, it remains to be shown that the $\mathscr{O}\left(n^2\xi\right)$ does not affect the variance significantly. To do this, we recall that we may approximate $\bar{x}_i \approx \mu_x \Phi_i$ and concentrate on estimating

$$\mathrm{Cov}(\Phi_i, \Phi_j) \quad \approx \quad \frac{1}{\mu_x^2}\mathrm{Cov}(\bar{x}_i, \bar{x}_j).$$

To do this, we make use of the following lemma and corollary.

Lemma 7. *Let X and Y be random variables and $Y \sim Bernoulli(p)$. Then $\mathbb{E}(X|Y=1) = \mathbb{E}(X) + \frac{1}{p}\mathrm{Cov}(X,Y)$.*

Proof. It is

$$\begin{aligned}
\mathbb{E}(XY) &= \mathbb{E}(\mathbb{E}(XY|Y)) \\
&= \mathbb{E}(Y\mathbb{E}(X|Y)) \\
&= p \cdot 1 \cdot \mathbb{E}(X|Y=1) + (1-p) \cdot 0 \cdot \mathbb{E}(X|Y=0) \\
&= p\mathbb{E}(X|Y=1).
\end{aligned}$$

On the other hand,

$$\begin{aligned}
\mathbb{E}(XY) &= \mathbb{E}(X)\mathbb{E}(Y) + \mathrm{Cov}(X,Y) \\
&= p\mathbb{E}(X) + \mathrm{Cov}(X,Y).
\end{aligned}$$

Consequently,

$$\mathbb{E}(X|Y=1) \quad = \quad \mathbb{E}(X) + \frac{1}{p}\mathrm{Cov}(X,Y)$$

as claimed. □

Corollary 4. *Let X, Y, and Z be random variables and $Z \sim Bernoulli(p)$. Then*

$$\begin{aligned}
\mathrm{Cov}(X,Y|Z=1) &= \mathrm{Cov}(X,Y) + \frac{1}{p}\big(\mathrm{Cov}(XY,Z) - \mathbb{E}(X)\mathrm{Cov}(Y,Z) \\
&\qquad -\mathbb{E}(Y)\mathrm{Cov}(X,Z) - \frac{1}{p}\mathrm{Cov}(X,Z)\mathrm{Cov}(Y,Z)\big) \\
\mathbb{V}(X|Z=1) &= \mathbb{V}(X) + \frac{1}{p}\big(\mathrm{Cov}\big(X^2,Z\big) - 2\mathbb{E}(X)\mathrm{Cov}(X,Z) - \frac{1}{p}\mathrm{Cov}(X,Z)^2\big)
\end{aligned}$$

Proof. The covariance formula follows from inserting the result of Lemma 7 into

$$\begin{aligned}
\mathrm{Cov}(X,Y|Z=1) &= \mathbb{E}(XY|Z=1) - \mathbb{E}(X|Z=1)\mathbb{E}(Y|Z=1) \\
&= \mathbb{E}(XY) + \frac{1}{p}\mathrm{Cov}(XY,Z) - \left(\mathbb{E}(X) + \frac{1}{p}\mathrm{Cov}(X,Z)\right)\left(\mathbb{E}(Y) + \frac{1}{p}\mathrm{Cov}(Y,Z)\right) \\
&= \mathbb{E}(XY) - \mathbb{E}(X)\mathbb{E}(Y) + \frac{1}{p}\mathrm{Cov}(XY,Z) - \frac{1}{p}\mathbb{E}(X)\mathrm{Cov}(Y,Z) \\
&\qquad -\frac{1}{p}\mathbb{E}(Y)\mathrm{Cov}(X,Z) - \frac{1}{p^2}\mathrm{Cov}(X,Z)\mathrm{Cov}(Y,Z) \\
&= \mathrm{Cov}(X,Y) + \frac{1}{p}\big(\mathrm{Cov}(XY,Z) - \mathbb{E}(X)\mathrm{Cov}(Y,Z) \\
&\qquad -\mathbb{E}(Y)\mathrm{Cov}(X,Z) - \frac{1}{p}\mathrm{Cov}(X,Z)\mathrm{Cov}(Y,Z)\big)
\end{aligned}$$

and the variance formula follows from $\mathbb{V}(X|Z) = \mathrm{Cov}(X,X|Z)$. □

Rearranging the equation in Lemma 7 leads to

$$\mathrm{Cov}(\Phi_i,\Phi_j) \;=\; P\left(\mathbb{E}\left(\Phi_i=1\middle|\Phi_j=1\right)-\mathbb{E}(\Phi_i)\right) \;=\; \varepsilon P,$$

or equivalently

$$\xi \quad = \quad \mu_x^2 P\varepsilon$$

with $\varepsilon := \mathbb{P}\left(\Phi_i=1\middle|\Phi_j=1\right)-\mathbb{P}(\Phi_i=1)$ being the difference between the probability that a species survives given that another species survives and the unconditioned probability that a species survives.

We argue that $|\varepsilon|$ is small if n is large. If

$$\begin{aligned}\mathbb{P}(\Phi_i=1) \quad &= \quad \mathbb{P}(\bar{g}_i\geq 0)\\ &= \quad \mathbb{P}\left(r_i+\sum_{j\in I_i}a_{ji}\bar{x}_j\geq 0\right)\end{aligned}$$

and

$$r_i+\sum_{j\in I_i}a_{ji}\bar{x}_j \quad \overset{d}{\approx} \quad \mathrm{N}\left(\mu_g,\,\sigma_g^2\right)$$

as approximated in the main text, we need to determine for some $j\neq i$

$$\varepsilon \quad = \quad \mathbb{P}\left(\bar{g}_i\geq 0\middle|\Phi_j=1\right)-\mathbb{P}(\bar{g}_i\geq 0).$$

Ignoring terms with lower orders of n, it is

$$\begin{aligned}\mathbb{E}(\bar{g}_i) \quad &\approx \quad \mu_r+(n-1)\,c\mu_a P\mu_x\\ &\approx \quad nc\mu_a P\mu_x\end{aligned}$$

and

$$\begin{aligned}\mathbb{V}(\bar{g}_i) \quad &\approx \quad \sigma_r^2+(n-1)\,cP\mu_x\left(\frac{1}{2}\,(1+\mu_x)\left(\mu_a^2+\sigma_a^2\right)-cP\mu_x\mu_a^2\right)+(n-2)\,(n-1)\,c^2\mu_a^2\xi+n\zeta\\ &\approx \quad n\left(cP\mu_x\left(\frac{1+\mu_x}{2}\left(\mu_a^2+\sigma_a^2\right)-cP\mu_x\mu_a^2\right)+\zeta\right)+n^2c^2\mu_a^2\xi\end{aligned}$$

with ζ being the constant representing the $\mathscr{O}\left(\frac{1}{n}\right)$ terms in $\mathrm{Cov}(a_{ji}x_j,a_{ki}x_k)$. Furthermore,

$$\begin{aligned}\mathbb{E}\left(\bar{g}_i\middle|\Phi_j=1\right) \quad &\approx \quad \mathbb{E}\left(r_i\middle|\Phi_j=1\right)+(n-2)\,\mathbb{E}\left(a_{ki}\bar{x}_k\middle|\Phi_j=1\right)+\mathbb{E}\left(a_{ji}\middle|\Phi_j=1\right)\\ &\approx \quad n\left(\mathbb{E}(a_{ki}\bar{x}_k)+\frac{1}{P}\mathrm{Cov}(a_{ki}\bar{x}_k,\Phi_j)\right)\\ &\approx \quad n\left(c\mu_a P\mu_x+\frac{c\mu_a\xi}{P\mu_x}\right).\end{aligned}$$

Here, we again applied Lemma 7 and neglected higher moments. Before approximating the conditional variance $\mathbb{V}\left(\bar{g}_i\middle|\Phi_j=1\right)$

note that

$$
\begin{aligned}
\mathrm{Cov}\left(r_i, a_{ki}\bar{x}_k \middle| \Phi_j = 1\right) &= \mathrm{Cov}(r_i, a_{ki}\bar{x}_k) + \frac{1}{P}\Bigg(\mathrm{Cov}(r_i a_{ki}\bar{x}_k, \Phi_j) - \mathbb{E}(r_i)\mathrm{Cov}(a_{ki}\bar{x}_k, \Phi_j) \\
&\quad - \mathbb{E}(a_{ki}\bar{x}_k)\mathrm{Cov}(r_i, \Phi_j) - \frac{1}{P}\mathrm{Cov}(r_i, \Phi_j)\mathrm{Cov}(a_{ki}\bar{x}_k, \Phi_j)\Bigg) \\
&= \mathscr{O}\left(\frac{1}{\sqrt{n}} + \xi\right),
\end{aligned}
$$

$$
\begin{aligned}
\mathrm{Cov}\left(a_{ji}, a_{ki}\bar{x}_k \middle| \Phi_j = 1\right) &= \mathrm{Cov}(a_{ji}, a_{ki}\bar{x}_k) + \frac{1}{P}\Bigg(\mathrm{Cov}(a_{ji} a_{ki}\bar{x}_k, \Phi_j) - \mathbb{E}(a_{ji})\mathrm{Cov}(a_{ki}\bar{x}_k, \Phi_j) \\
&\quad - \mathbb{E}(a_{ki}\bar{x}_k)\mathrm{Cov}(a_{ji}, \Phi_j) - \frac{1}{P}\mathrm{Cov}(a_{ji}, \Phi_j)\mathrm{Cov}(a_{ki}\bar{x}_k, \Phi_j)\Bigg) \\
&= \mathscr{O}\left(\frac{1}{\sqrt{n}} + \xi\right),
\end{aligned}
$$

$$
\begin{aligned}
\mathrm{Cov}\left(a_{ki}^2\bar{x}_k^2, \Phi_j\right) &= \mathrm{Cov}\left(a_{ki}^2\bar{x}_k, \Phi_j\right) \\
&= \mathscr{O}\left(\frac{1}{n} + \xi\right),
\end{aligned}
$$

$$
\mathrm{Cov}(a_{ki}\bar{x}_k, \Phi_j) = \mathscr{O}\left(\frac{1}{n} + \xi\right),
$$

and

$$
\begin{aligned}
\mathrm{Cov}(a_{ki}\bar{x}_k a_{li}\bar{x}_l, \Phi_j) &\approx 2\mathbb{E}(a_{ki}\bar{x}_k)\mathrm{Cov}(a_{li}\bar{x}_l, \Phi_j) \\
&\approx 2\left(c\mu_a P\mu_x + \mathrm{Cov}(a_{ki}, \bar{x}_k)\right)\left(c\mu_a \mathrm{Cov}(\bar{x}_l, \Phi_j) + P\mu_x \mathrm{Cov}(a_{li}, \Phi_j)\right) \\
&= 2\left(c\mu_a P\mu_x + \mathscr{O}\left(\frac{1}{\sqrt{n}}\right)\right)\left(\frac{c\mu_a\xi}{\mu_x} + \mathscr{O}\left(\frac{1}{n}\right)\right) \\
&= 2c^2\mu_a^2 P\xi + \mathscr{O}\left(\frac{\xi}{\sqrt{n}} + \frac{1}{n}\right). \qquad \text{(S25)}
\end{aligned}
$$

Note that $|\xi| < 1$ and therefore $\mathscr{O}\left(\xi + \xi^2\right) = \mathscr{O}(\xi)$. With equation (S25), we may write

$$
\mathrm{Cov}(a_{ki}\bar{x}_k a_{li}\bar{x}_l, \Phi_j) - 2\mathbb{E}(a_{ki}\bar{x}_k)\mathrm{Cov}(a_{ki}\bar{x}_k, \Phi_j) = c^2\mu_a^2 P\nu\xi, \qquad \text{(S26)}
$$

whereby ν is a small constant accounting for the higher moments initially neglected in equation (S25). The reason to consider higher moments here is that the terms dominating higher moments in $\mathrm{Cov}(a_{ki}\bar{x}_k a_{li}\bar{x}_l, \Phi_j)$ and $2\mathbb{E}(a_{ki}\bar{x}_k)\mathrm{Cov}(a_{ki}\bar{x}_k, \Phi_j)$ cancel each other out in (S26). It is reasonable to assume that $\nu \in [-1, 1]$. As will be apparent later, $\nu < -1$ would result in a negative

variance. With these observations, we can estimate the conditional variance for large n:

$$\begin{aligned}
\mathbb{V}\left(\bar{g}_i \middle| \Phi_j = 1\right) &\approx \mathbb{V}\left(r_i \middle| \Phi_j = 1\right) + (n-2)\,\mathbb{V}\left(a_{ki}\bar{x}_k \middle| \Phi_j = 1\right) + \mathbb{V}\left(a_{ji} \middle| \Phi_j = 1\right) \\
&\quad +2\,(n-2)\left(\mathrm{Cov}\left(r_i, a_{ki}\bar{x}_k \middle| \Phi_j = 1\right) + \mathrm{Cov}\left(a_{ji}, a_{ki}\bar{x}_k \middle| \Phi_j = 1\right)\right) \\
&\quad +(n-3)\,(n-2)\,\mathrm{Cov}\left(a_{ki}\bar{x}_k, a_{li}\bar{x}_l \middle| \Phi_j = 1\right). \\
&\approx n\mathbb{V}(a_{ki}\bar{x}_k) + \frac{n}{P}\left(\mathrm{Cov}\left(a_{ki}^2\bar{x}_k^2, \Phi_j\right) - 2\mathbb{E}(a_{ki}\bar{x}_k)\mathrm{Cov}(a_{ki}\bar{x}_k, \Phi_j) - \frac{1}{P}\mathrm{Cov}(a_{ki}\bar{x}_k, \Phi_j)^2\right) \\
&\quad +n^2\left(\mathrm{Cov}(a_{ki}\bar{x}_k, a_{li}\bar{x}_l) + \frac{1}{P}\left(\mathrm{Cov}(a_{ki}\bar{x}_k a_{li}\bar{x}_l, \Phi_j) - 2\mathbb{E}(a_{ki}\bar{x}_k)\mathrm{Cov}(a_{ki}\bar{x}_k, \Phi_j) - \frac{1}{P}\mathrm{Cov}(a_{ki}\bar{x}_k, \Phi_j)^2\right)\right) \\
&\approx n\left(cP\mu_x\left(\frac{1+\mu_x}{2}\left(\mu_a^2 + \sigma_a^2\right) - cP\mu_x\mu_a^2\right) + \zeta\right) + n^2c^2\mu_a^2\xi(1+\nu)
\end{aligned}$$

Now we can attempt to estimate ξ for large n. Let us first assume that $\xi > \mathscr{O}\left(\frac{1}{n}\right)$. Then the n^2 terms dominate the variance and we obtain

$$\begin{aligned}
\frac{\xi}{P\mu_x^2} &\approx S_\mathrm{N}\left(0, nc\mu_a P\mu_x + n\frac{c\mu_a\xi}{P\mu_x}, n^2c^2\mu_a^2\xi(1+\nu)\right) - S_\mathrm{N}\left(0, nc\mu_a P\mu_x, n^2c^2\mu_a^2\xi\right) \\
&= \mathrm{sign}\left(\mu_a\right)\left(S_\mathrm{N}\left(0, P\mu_x + \frac{\xi}{P\mu_x}, \xi(1+\nu)\right) - S_\mathrm{N}\left(0, P\mu_x, \xi\right)\right). \qquad \text{(S27)}
\end{aligned}$$

If $\mu_a = 0$, it follows directly that $\xi = 0$, which contradicts our claim $\xi > \mathscr{O}\left(\frac{1}{n}\right)$. Hence, we consider $\mu_a \neq 0$. Let $\tilde{\xi} := \frac{\xi}{P\mu_x^2}$. Then

$$\begin{aligned}
\mathrm{sign}\left(\mu_a\right)\tilde{\xi} &= S_\mathrm{N}\left(0, P\mu_x + \tilde{\xi}\mu_x, \tilde{\xi}P\mu_x^2(1+\nu)\right) - S_\mathrm{N}\left(0, P\mu_x, \tilde{\xi}P\mu_x^2\right) \\
&= S_\mathrm{N}\left(0, P + \tilde{\xi}, \tilde{\xi}P(1+\nu)\right) - S_\mathrm{N}\left(0, P, \tilde{\xi}P\right) \qquad \text{(S28)}
\end{aligned}$$

under the constraint

$$P = S_\mathrm{N}\left(0, \mathrm{sign}\left(\mu_a\right)P, \tilde{\xi}P\right). \qquad \text{(S29)}$$

We argue that this system of equations does not have a "significant" solution for reasonable choices of ν. Let us first note that $\xi > 0$, because $\mathbb{V}(\bar{g}_i)$ would turn negative for large n otherwise. Observe that since $P, \tilde{\xi} > 0$, the term $S_\mathrm{N}\left(0, P + \tilde{\xi}, \tilde{\xi}P(1+\nu)\right)$ is decreasing as ν is increasing. We know that $\nu \geq -1$. Hence,

$$\begin{aligned}
\tilde{\xi} &= S_\mathrm{N}\left(0, P + \tilde{\xi}, \tilde{\xi}P(1+\nu)\right) - S_\mathrm{N}\left(0, P, \tilde{\xi}P\right) \\
&< S_\mathrm{N}\left(0, P + \tilde{\xi}, 0\right) - S_\mathrm{N}\left(0, P, \tilde{\xi}P\right) \\
&= 1 - S_\mathscr{N}\left(0, P, \tilde{\xi}P\right) \\
&= F_\mathrm{N}\left(0, \sqrt{P}, \tilde{\xi}\right). \qquad \text{(S30)}
\end{aligned}$$

F_N is the cumulative density function of the normal distribution. It is a simple exercise to show that $F_\mathrm{N}\left(0, \sqrt{P}, \tilde{\xi}\right)$ is decreasing if P increases. A plot of $F_\mathrm{N}\left(0, \sqrt{P}, \tilde{\xi}\right) - \tilde{\xi}$ for $P = 0.2$ shows that (S30) does not have a solution for $P \geq 0.2$. That is, if P becomes sufficiently large, then our assumption $\xi > \mathscr{O}\left(\frac{1}{n}\right)$ must be wrong. Since $\mu_a > 0$ implies that the mean value μ_g is positive for large n (see equation (S29)), it must be $\xi = \mathscr{O}\left(\frac{1}{n}\right)$ if $\mu_a > 0$.

Now let us consider the case of $\mu_a < 0$. Under this condition, equation system (S28)-(S29) does have a solution, which can be determined numerically for each given ν. We have already noted that $S_\mathrm{N}\left(0, P + \tilde{\xi}, \tilde{\xi}P(1+\nu)\right)$ is decreasing in ν. Consequently, $\tilde{\xi}$ and therefore ξ is maximized if ν is chosen to be large. If $\nu \leq 1$ as deemed reasonable above, then the largest

ξ value we can obtain is attained with $\nu = 1$. Solving system (S28)-(S29) numerically yields $\xi \approx 4.3 \cdot 10^{-4}\mu_x$. For $\nu = 0.5$, we obtain $\xi \approx 2.2 \cdot 10^{-5}\mu_x$ and for $\nu \leq 0$, the system does not have a solution. That is, if ξ does not vanish, it becomes important in large systems (1000 species in order of magnitude) only, and only if higher order moments are of same order of magnitude as lower order moments. This observation and numerical simulations (see Appendix E) show that our heuristic will do well in the systems we considered in this study.

In summary, the variance approximation

$$\mathbb{V}(\bar{g}_i) \quad = \quad \sigma_r^2 + (n-1)\, cP\mu_x \left(\frac{1+\mu_x}{2} \left(\mu_a^2 + \sigma_a^2\right) - cP\mu_x\mu_a^2 \right) \tag{S31}$$

is valid whenever $\mu_a \geq 0$ and a reasonable approximation for $\mu_a < 0$, unless n is very large.

Remark. We conducted all the computations above under the assumption that there is a unique estimate for P. However, in systems that exhibit "persistence bistability", i.e. P depends on the initial condition, $\Phi_j = 1$ may imply a move from one persistence state to another. As a consequence, it is possible that in such systems either all species survive or all species die; i.e. $\xi \approx \mu_x^2 P(1-P)$. In this case, our persistence estimates can be far from the actual persistence values. Therefore, our heuristic performs poorly in the direct neighbourhood of its jump discontinuities. The size of this neighbourhood depends on the variance of the initial condition. This can be observed in Figure 3e in the main text, where our heuristic performs particularly poorly in the case with 60% invaders (an initial condition with large variance). Note also the respective figures in Appendix E.

E Numerical estimates of unknown quantities crucial for the derivation of the heuristic

Our derivation of a heuristic for the expected portion P of persisting species requires a number of assumptions:

1. The conditional mean $\mu_x = \mathbb{E}(\bar{x}_i | \bar{x}_i > 0)$ is close to 1.
2. The conditional variance $\sigma_x^2 = \mathbb{V}(\bar{x}_i | \bar{x}_i > 0)$ is small and reasonably well approximated by $\sigma_x^2 \approx \frac{1}{2}\mu_x(1-\mu_x)$.
3. The variance $\sigma_P^2 = \mathbb{V}(\Psi)$ is small. Here, $\Psi := \frac{1}{n}\sum_{i \in I} \mathrm{I}_{(0,1]}(\bar{x}_i)$ is the random variable describing the proportion of surviving species; $P = \mathbb{E}(\Psi)$.
4. The covariance $\xi = \mathrm{Cov}(\bar{x}_i, \bar{x}_j)$, $i \neq j$ is small.

In the main text, we have provided arguments supporting these claims, and in Appendix C, we have shown that the third claim is true for large systems. However, we did not provide any quantitative estimates for the approximated quantities. In this appendix, we present numerical estimates for μ_x, σ_x^2, and ξ and add a short discussion of these results regarding their implications for our heuristic.

As the purpose of this appendix is to confirm that the approximations made in the main text and Appendix C are valid, we need to show that the ξ term does not play a dominant role in the variance estimate

$$\begin{aligned}
\mathbb{V}(\bar{g}_i) \quad &\approx \quad n\left(cP\mu_x^2\left(\mu_a^2(1-Pc) + \sigma_a^2\right) + \zeta\right) + n^2c^2\mu_a^2\xi \\
&= \quad n\left(cP\mu_x^2\left(\mu_a^2(1-Pc) + \sigma_a^2\right) + \zeta + nc^2\mu_a^2\xi\right).
\end{aligned}$$

Therefore, it makes sense to consider the quantity $n\xi$, as it is crucial that this product is bounded. Therefore, we will present the covariance results in terms of the quantity $n\xi$ below.

E.1 Method

To estimate any of the quantities of interest numerically, we need a sufficiently large sample of long-time average densities $\bar{\mathbf{x}}$. We computed this sample with the same method described in the section 'Simulation method' in the main text. We drew the random model parameters according to their respective distributions and simulated the resulting systems for $T = 1000$ time

steps. To avoid a bias due to the "lock-in phase", in which the species densities are far from their average, we determined $\bar{\mathbf{x}}$ by averaging the species densities in the time interval $\left[\frac{3}{4}T, T\right]$. We repeated this procedure $N = 1000$ times. To save computing time, we used the same $\bar{\mathbf{x}}$ sample here that we also used to obtain the results for P presented in the main text.

Let $\bar{\mathbf{x}}^{(k)}$ be the vector of average species densities in the kth simulation run. To estimate μ_x, we simply took the average of all $\bar{x}_i^{(k)}$, $i \in I := \{1, \ldots, n\}$, with $\bar{x}_i^{(k)} > \varepsilon = 0.001$, whereby epsilon was the extinction threshold described in the main text. That is

$$\mu_x \approx \frac{1}{\tilde{N}} \sum_{k=1}^{N} \sum_{i \in I : \bar{x}_i^{(k)} \geq \varepsilon} \bar{x}_i^{(k)}$$

with

$$\tilde{N} = \sum_{k=1}^{N} \sum_{i \in I : \bar{x}_i^{(k)} \geq \varepsilon} 1.$$

The conditional variance σ_x^2 can can be computed accordingly:

$$\begin{aligned} \sigma_x^2 &:= \mathbb{E}\left(\bar{x}_i^2 | \bar{x}_i > 0\right) - \mathbb{E}(\bar{x}_i | \bar{x}_i > 0)^2 \\ &\approx \frac{1}{\tilde{N}} \sum_{k=1}^{N} \sum_{i \in I : \bar{x}_i^{(k)} \geq \varepsilon} \left(\bar{x}_i^{(k)}\right)^2 - \mu_x^2. \end{aligned}$$

In the same manner, we computed the variance σ_P^2:

$$\begin{aligned} \sigma_x^2 &:= \mathbb{E}\left(\Psi^2\right) - \mathbb{E}(\Psi)^2 \\ &\approx \frac{1}{N} \sum_{k=1}^{N} \left(\frac{1}{n} \sum_{i \in I : \bar{x}_i^{(k)} \geq \varepsilon} 1 \right)^2 - \left(\frac{1}{Nn} \sum_{k=1}^{N} \sum_{i \in I : \bar{x}_i^{(k)} \geq \varepsilon} 1 \right)^2. \end{aligned}$$

The covariance ξ is defined as

$$\xi := \mathbb{E}(\bar{x}_i \bar{x}_j) - \mathbb{E}(\bar{x}_i)\mathbb{E}(\bar{x}_j).$$

We computed the expected value $\mathbb{E}(\bar{x}_i) = \mathbb{E}(\bar{x}_j)$ by taking the average over all $\bar{x}_i^{(k)}$; $i \in I$, $k \in \{1, \ldots, N\}$. To compute the expected value $\mathbb{E}(\bar{x}_i \bar{x}_j)$ of the product of different average species densities, we determined for each $k \in \{1, \ldots, N\}$ and each $i \in I$, $j \in I_i := I \setminus \{i\}$ the product $\phi_{ij}^{(k)} := \bar{x}_i^{(k)} \bar{x}_j^{(k)}$ and computed the mean of the results. In summary,

$$\xi \approx \frac{1}{Nn(n-1)} \sum_{k=1}^{N} \sum_{i \in I} \sum_{j \in I_i} \bar{x}_i^{(k)} \bar{x}_j^{(k)} - \left(\frac{1}{Nn} \sum_{k=1}^{N} \sum_{i \in I} \bar{x}_i^{(k)} \right)^2.$$

A main result of this paper is that the behaviour of the complete system can be deduced from looking at an "average" species. Therefore, the proportion of persisting species is driven by the number of links per species. It is reasonable to hypothesize that the same applies to the quantities considered in this Appendix. Therefore, we will present our numerical results in relation to the linkage density $l = c(n-1)$. Also, this makes it easy to link the results presented in this appendix with the results presented in the main text.

E.2 Results

In this section, we briefly describe the simulation results that we obtained.

The results for the average density μ_x of surviving species is shown in figure S1. The density of surviving species is mainly

driven by the linkage density l. For multiple combinations of the parameters μ_r, σ_r^2, μ_a, and σ_a^2, we see a steep initial ascent of the μ_x curve, which then levels out. Except for the case with strongly negatively biased interactions (Figure S1d) and the bistable case (Figure S1f), the initial densities of the species do not affect μ_x strongly. In systems with strong damping (Figure S1e), the average density of surviving species is small. In these systems, μ_x could be estimated with high accuracy.

Figure S2 shows the variance σ_x^2 of the average densities of surviving species. Similar to the mean μ_x, the variance of the average densities is driven by the linkage density. For some parameter combinations, the curves increase for small l. The larger the linkage density gets, however, the closer the curves approach a limiting value, which is often small. The initial densities have a small effect on σ_x^2 except for the bistable case (Figure S2e) and systems with strongly negatively biased interactions (Figure S2d). In the latter case, the curve for the colonization scenario is qualitatively different to the respective curve for the scenario with high initial densities.

In most systems, the estimate of the variance based on the value of the mean μ_x yielded good results. Though quantitative deviations are visible, the estimates had the right order of magnitude and had a qualitatively similar dependency on l as the computed exact variances.

In Figure S3, we present simulation results for the variance σ_P^2 of the proportion of coexisting species. In most systems, there is no connection between the linkage density and σ_P^2 visible. However, in all simulated scenarios, σ_P^2 was very small in systems with large linkage density. In systems with persistence bistability (Figure S3e), the variance has a sharp spark in a neighbourhood of the jump discontinuity of the heuristic. The sharpness of the spike is related to the variance of the distribution of initial densities: the region of large σ_P^2 is widest in the scenario with some invaders (low density) and some native species (high density).

The simulation results for the scaled inter-species correlation $n\xi$ are displayed in Figure S4. For some parameter combinations, we see a clear relationship between $n\xi$ and the linkage density l. However, in other systems, the values are more scattered. In all considered systems, $n\xi$ approaches a respective limit value below 0.5. The covariance is positive in all systems, even in those in which species affect each other negatively on average (Figures S4b and S4d). The quantity $n\xi$ becomes large in the systems with persistence bistability only (Figure S4e). The covariance peaks around the jump discontinuity of our heuristic. The spike is widest in scenarios with highly variant initial densities.

E.3 Discussion

Most of the quantities considered in this appendix are strongly dependent on the linkage density l. This suggests that it may be possible to derive heuristic estimates for these quantities in future and thereby refine the heuristic presented. Regardless of this potential for future improvement, most of the approximations our heuristic is based on turned out to be valid.

As assumed for the derivation of the heuristic, the mean average density μ_x of surviving species is large and mostly constant for systems without strong intra-specific competition. In systems with strong self-damping (Figure S1e), the heuristic estimates are highly accurate. The results in Figure S1f are scattered for small l due to the small sample size of persistent species, as surviving species are rare in systems with small persistence values P. Of higher concern are the initial rapid changes of μ_x with l found in all other simulated systems. This change could make our heuristic imprecise. Note, however, that the distribution of the growth constants is the main driver of persistence in systems with small linkage density. That is, in systems with small linkage density, μ_x has only a minor effect on persistence. Therefore, our heuristic approximation based on a constant and large value of μ_x performs well in general.

To reduce model complexity, we approximated of the variance σ_x^2 of the average densities of surviving species using the corresponding mean μ_x. This approximation turned out to yield valuable estimates. Though the approximation is not always quantitatively precise, the order of magnitude of σ_x^2 is well approximated. Different scaling factor than $\omega_1 = \frac{1}{2}$ and $\omega_2 = 2$ for the estimates $\sigma_x^2 \approx \omega_1 \mu_x (1 - \mu_x)$ and $\sigma_x^2 \approx \frac{\omega_2}{3} \mu_x^2$, respectively may yield even better estimates. However, the value of this constant would differ from case to case. As we do not know a mechanistic model for σ_x^2, we suggest to keep the (admittedly somewhat arbitrary) choice of "intermediate" $\omega_1 = \frac{1}{2}$ and $\omega_2 = 2$.

The smaller the variance σ_P^2 of the fraction of persisting species the more precise will our approximation of the mean of the

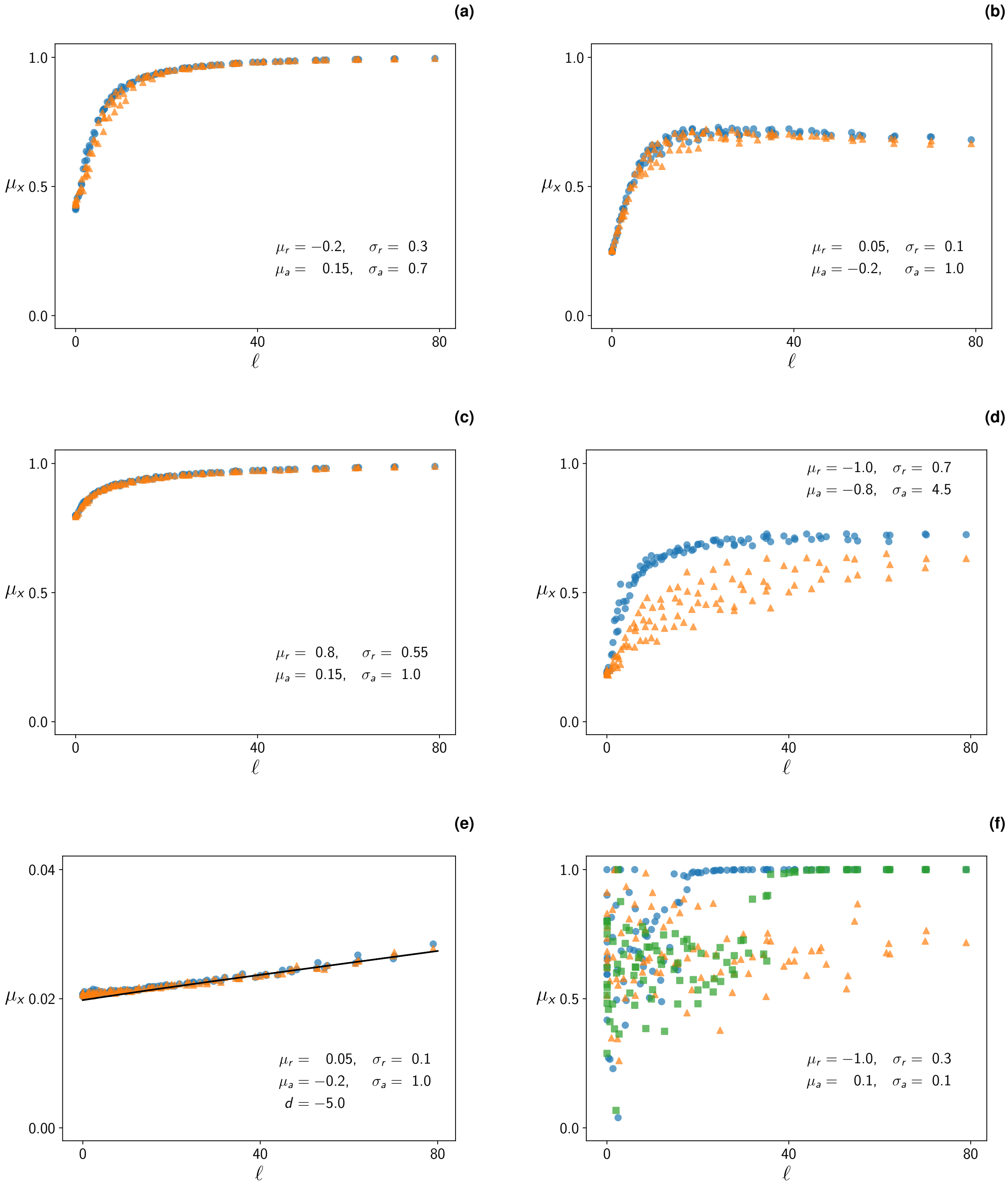

Figure S1. Mean long-time average density μ_x of persistent species as function of the linkage density l. Each subfigure corresponds to networks with different distributions of growth and interaction constants, respectively.
Each marker represents a network with specific species richness n and connectivity c. The blue dots refer to networks with high initial species densities ($b = 1$), and orange triangles to networks with very low initial densities (colonization scenario, $b = 0$). The green squares in Subfigure S1f depict an intermediate scenario with $b = 0.4$. Note that the estimates of μ_x are imprecise in networks in which only few species survive. Therefore, the markers in Subfigure S1f are scattered. In the scenario with strong damping (Subfigure S1e), specific estimates for μ_x could be computed. These estimates are plotted as a solid line.
It is visible that μ_x assumes high values in most networks except for those with strong damping (Subfigure S1e). Nonetheless, μ_x changes with the linkage density l.

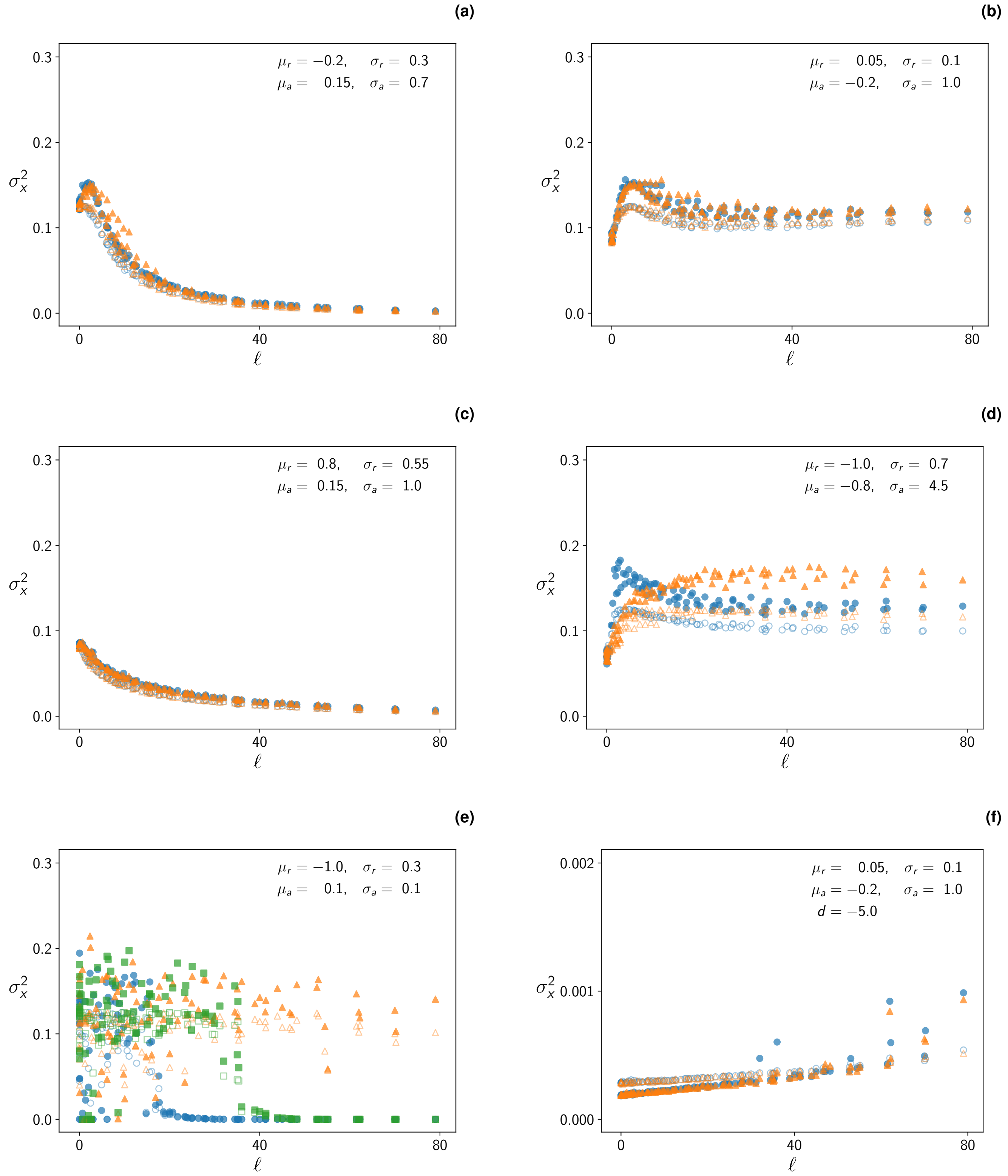

Figure S2. Variance σ_x^2 of the long-time average density of persistent species as function of the linkage density l. Each subfigure corresponds to networks with different distributions of growth and interaction constants, respectively.
Each marker represents a network with specific species richness n and connectivity c. The blue dots refer to networks with high initial species densities ($b = 1$), and orange triangles to networks with very low initial densities (colonization scenario, $b = 0$). The green squares in Fig. S1f depict an intermediate scenario with $b = 0.4$. The empty markers depict the variance estimates based on the average densities: $\hat{\sigma}_x^2 = \frac{2}{3}\mu_x^2$ in Fig. S2f and $\hat{\sigma}_x^2 = \frac{1}{2}\mu_x(1-\mu_x)$ in the remaining subfigures. Note that the numerical estimates of σ_x^2 are imprecise in networks in which only few species survive. Therefore, the markers in Fig. S2e are scattered.
It is visible that the variance estimate based on μ_x is reasonably precise. Furthermore, σ_x^2 is rather small in general.

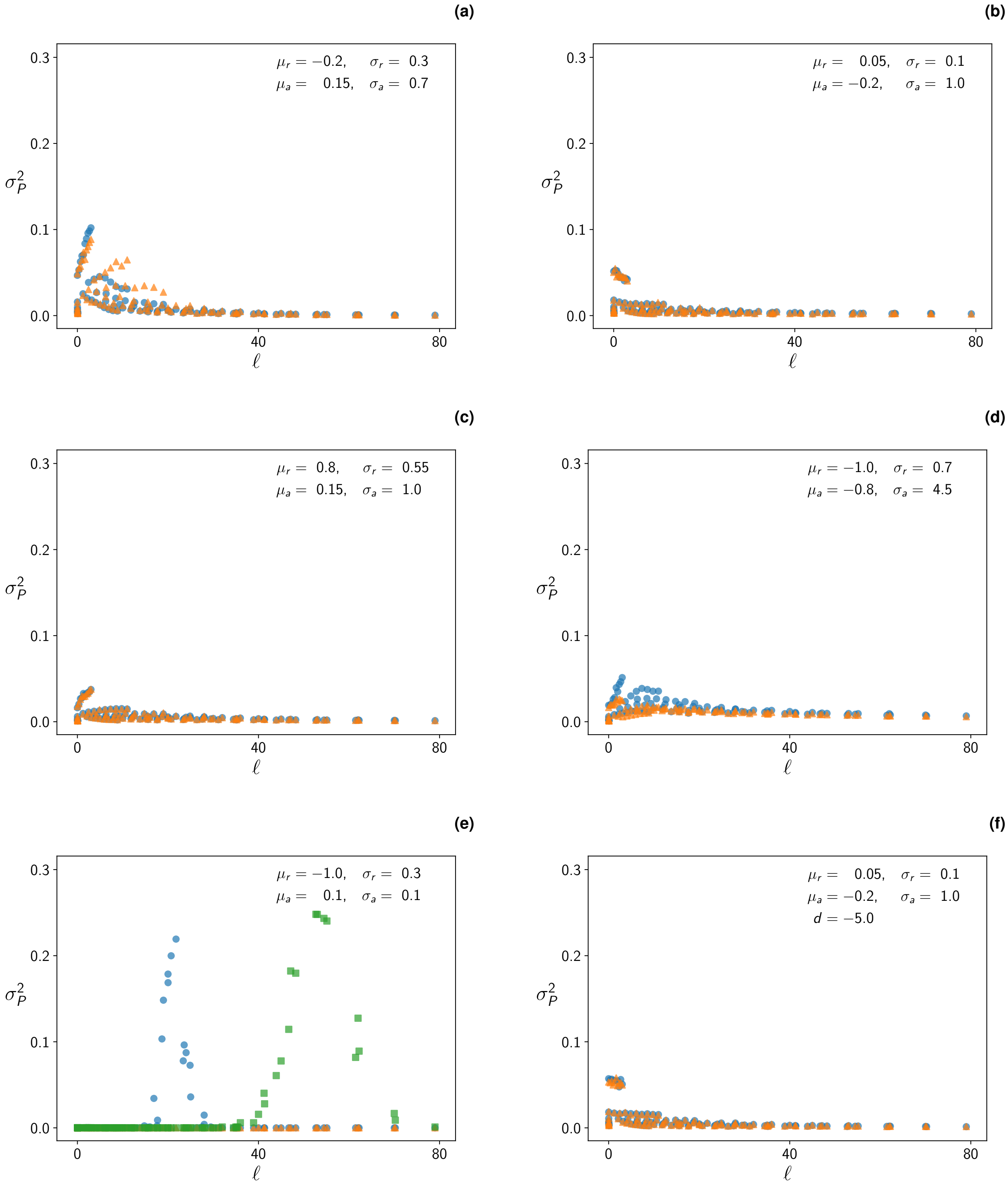

Figure S3. Variance σ_P^2 of the proportion of coexisting species as function of the linkage density l. Each subfigure corresponds to networks with different distributions of growth and interaction constants, respectively.
Each marker represents a network with specific species richness n and connectivity c. The blue dots refer to networks with high initial species densities ($b = 1$), and orange triangles to networks with very low initial densities (colonization scenario, $b = 0$). The green squares in Fig. S3e depict an intermediate scenario with $b = 0.4$.
Our heuristic requires that σ_P^2 is small if l is large and P is small. This is satisfied in general except for systems with persistence bistability (Figure S3e) where the condition is violated in the neighbourhood of the jump discontinuity of the persistence values. As σ_P^2 is strongly related to ξ, refer to the concluding remark D.3 of Appendix D.3 for an explanation of this system behaviour.

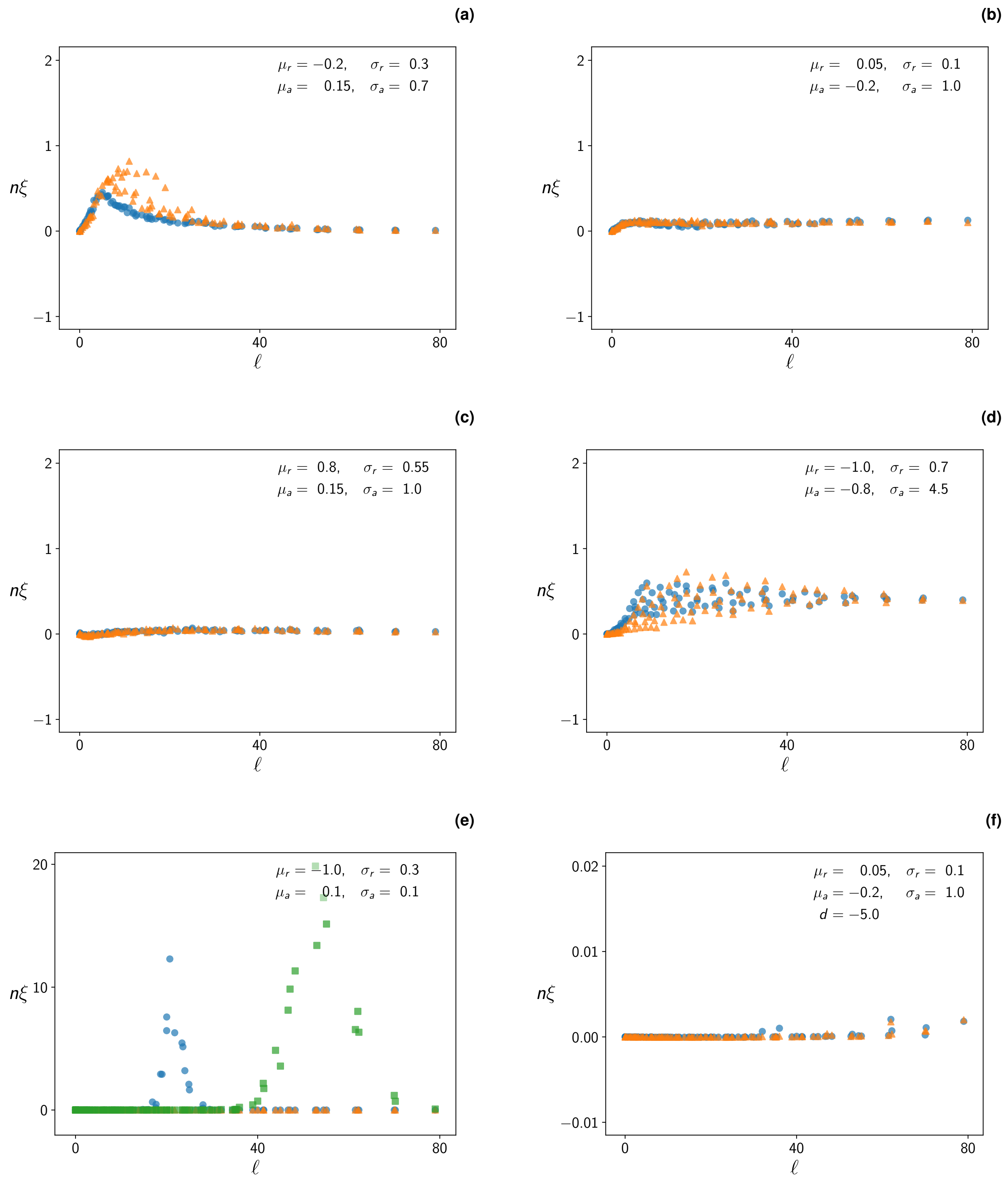

Figure S4. Covariance ξ between different species' average densities as function of the linkage density l. The covariances are displayed multiplied with the species richnesses of the respective networks, as this product appears in the variance estimate for the average per-capita growth rate $\bar{g}_i$. Our heuristic approximation requires that $n\xi$ is bounded. Each subfigure corresponds to networks with different distributions of growth and interaction constants, respectively.

Each marker represents a network with specific species richness n and connectivity c. The blue dots refer to networks with high initial species densities ($b = 1$), and orange triangles to networks with very low initial densities (colonization scenario, $b = 0$). The green squares in Fig. S4e depict an intermediate scenario with $b = 0.4$.

The covariance term is bounded by a small value as required by our heuristic. Only in the network with persistence bistability (Fig. S4e), the covariance term gets large around the jump point. We explained this behaviour in the concluding remark D.3 of Appendix D.3.

exponential term in our heuristic be. Though σ_P^2 is consistently small for systems with large linkage density, it turns our to be too large to make our heuristic precise even in large, highly linked systems with few persisting species.

The spark of σ_P^2 around jump points of the persistence value P in systems with persistence bistability can be well explained by the impact of the initial conditions on the result. Initial conditions have a strong impact on the behaviour of systems exhibiting persistence bistability. If the initial conditions are drawn from a distribution with large variance, it is as well possible that the initial conditions favour persistence as it is possible that they lead to a trajectory with many extinctions. The more the two possible persistence states are apart from each other the larger is the variance of the proportion of persisting species. In the most extreme case, either all species survive or all species go extinct. This can lead to the maximal possible variance $P(P-1)$.

Our heuristic for P is based on the assumption that the correlations between average species densities is small. In Appendix D we used an approximation to show that this requirement is met in the systems that we considered in this paper. Our numerical estimates support this claim. However, the correlation is large around persistence jump points in systems with persistence bistability. This does not contradict our argument for small covariances, as this effect is due to the randomness of the initial conditions as described above and in the concluding remark D.3 of Appendix D.3. Nonetheless, we can conclude that our heuristic is imprecise around jump points. The size of the region of imprecision depends on the variance of the initial conditions.

In summary, our numerical results support the claims and approximations made in the main paper. However, the results also reveal limitations of the heuristic in systems with persistence bistability.

F Supplementary information on Figure 1

In this Appendix, we provide supplementary information on the creation of Figure 1 (main text).

We used the same simulation approach as outlined in the section ‘Simulation method’ (main text) and Supplementary Appendix E to compute a sample of

$$\bar{g}_i^{(k)} \quad := \quad \sum_{j\in I_i} a_{ji}^{(k)} \bar{x}_i^{(k)}$$

for $i \in I := \{1,\dots,n\}$, $I_i := I\backslash\{i\}$, and $k \in \{1,\dots,10000\}$. Here, k enumerates the simulation runs. We computed $\bar{x}_i$ by averaging simulated population densities $x_i^{(k)}(t)$ over the time interval $\left[\frac{3}{4}T, T\right]$ with $T = 1000$. Since the purpose of Figure 1 is to depict the quality of our approximation of $\bar{g}_i$ given that species i is affected by at least one other species, we excluded all $\bar{g}_i^{(k)}$ with $\bar{g}_i^{(k)} = 0$ from our sample. We created normalized histograms of $G := \left\{\bar{g}_i^{(k)} | \bar{g}_i^{(k)} \neq 0\right\}$ for the two simulated systems. To depict the observed cumulative density function, we created cumulative histograms, respectively.

From our simulations, we computed the unknown parameters μ_x, σ_x^2, and P as described in the section ‘Simulation method’ (main text) and Supplementary Appendix E. Then we used the resulting values to compute the approximate mean μ_g and variance σ_g^2 according to equations (7) and (10) (main text), respectively. Thereby, we neglected covariance terms. Finally, we plotted the normal probability density function and cumulative density function with the obtained mean and variance in the corresponding figures.

We simulated systems with the following parameters: Subfigures 1a, b: $n = 60$, $c = 0.5$, $\mu_r = -1$, $\sigma_r = 0.7$, $\mu_a = -0.8$, $\sigma_a = 4.5$, $b = 1$; Subfigures 1c, d: $n = 60$, $c = 0.5$, $\mu_r = -0.2$, $\sigma_r = 0.3$, $\mu_a = 0.15$, $\sigma_a = 0.7$, $b = 1$.

G Sharp density bound and functional response

The model our study is based on involves a sharp threshold that limits the growth of each species i. It is

$$\frac{dx_i}{dt} \quad = \quad \begin{cases} x_i g_i(\mathbf{x}) & \text{if } x_i < 1 \text{ or } g_i(\mathbf{x}) < 0 \\ 0 & \text{else,} \end{cases} \tag{S32}$$

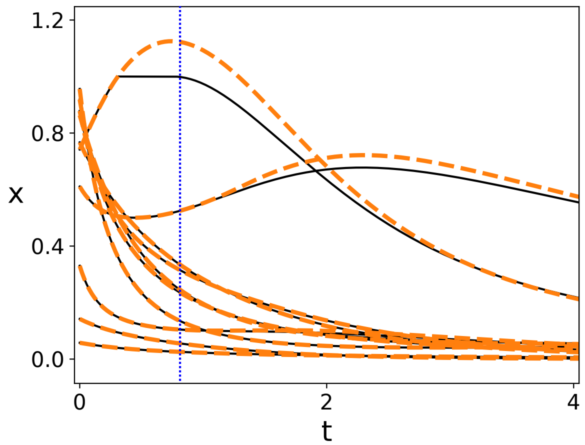

Figure S5. Comparison of the system (S32) with bounded species densities (solid black lines) and the system (S33) with bounded interaction terms (dashed orange lines). Until the first species i in system (S32) leaves the bounding density 1 (time point depicted as blue dotted vertical line), the trajectories of the two systems match exactly. After that time point, the reduced density of species i has a delayed effect in system (S33).

with $i \in I := \{1,\ldots,n\}$ and $g_i(\mathbf{x}) := r_i + \sum_{j\in I} a_{ji}x_j$. The introduction of a sharp threshold can be criticised as lacking ecological motivation. However, the qualitative dynamics of system (S32) with respect to persistence resemble the dynamics of a system with per-interaction functional response of Holling type one,

$$\frac{dx_i}{dt} \quad = \quad x_i\left(r_i + \sum_{j\in I_i} a_{ji}\min(x_j,1) + a_{ii}x_i\right). \tag{S33}$$

All results presented in this paper can be directly applied to system (S33) if the variables are redefined so that $\mu_x = \mathbb{E}\big(\min(x_i,1)\big|x_i > 0\big)$ and $\sigma_x^2 = \mathbb{V}\big(\min(x_i,1)\big|x_i > 0\big)$. Though proper introduction of functional response would require that the minimum function in system (S33) would include the sum over all prey species, and the interactions with predators should be adjusted accordingly, the dynamics of system (S33) may provide some hints on how a system with legitimate functional response would behave qualitatively.

Though the proof of the above claim is done by applying the steps of our heuristic derivation to system (S33) with the given main adjustments, we can gain a better understanding of why the model change does not affect persistence by comparing the dynamics of the two systems (see Figure S5). Note that the dynamics of systems (S32) and (S33) are similar until a species in system (S32) leaves the threshold density for the first time. Until this point in time, the densities of the species in the two systems are equal except for species that reach the threshold in system (S32). These species will continue to grow in system (S33), though this will not change the effect of these species on other species due to the functional response. The difference between the systems appears when the growth of a species i with density $x_i \geq 1$ turns negative for the first time. While this change push the density of species i below 1 in system (S32) immediately, which directly affects the impact of species i on other species, the transition to a density below the threshold will occur later in system (S33). Hence, system (S33) can be understood as a delayed version of system (S32). It is intuitive that this delay does not have a strong effect on persistence.

References

1. Cortés, J. Discontinuous dynamical systems. *IEEE Control. Syst.* **28**, 36–73, DOI: 10.1109/MCS.2008.919306 (2008).

2. Filippov, A. F. *Differential Equations with Discontinuous Righthand Sides*, vol. 18 of *Mathematics and Its Applications* (Springer Netherlands, Dordrecht, 1988).

3. Bohrnstedt, G. W. & Goldberger, A. S. On the Exact Covariance of Products of Random Variables. *J. Am. Stat. Assoc.* **64**, 1439–1442, DOI: 10.2307/2286081 (1969).
